# Trade-Offs Predicted by Metabolic Network Structure Give Rise to Evolutionary Specialization and Phenotypic Diversification

**DOI:** 10.1093/molbev/msac124

**Published:** 2022-06-09

**Authors:** David M Ekkers, Sergio Tusso, Stefany Moreno-Gamez, Marina C Rillo, Oscar P Kuipers, G Sander van Doorn

**Affiliations:** Groningen Institute for Evolutionary Life Sciences (GELIFES), University of Groningen, Nijenborgh 7, 9747 AG Groningen, The Netherlands; Molecular Genetics Group, Groningen Biomolecular Sciences and Biotechnology Institute, University of Groningen, Nijenborgh 7, 9747 AG Groningen, The Netherlands; Division of Evolutionary Biology, Faculty of Biology, LMU Munich, Grosshaderner Str. 2, 82152 Planegg-Martinsried, Germany; Science for Life Laboratories and Department of Evolutionary Biology, Norbyvägen 18D, Uppsala University, 75236 Uppsala, Sweden; Groningen Institute for Evolutionary Life Sciences (GELIFES), University of Groningen, Nijenborgh 7, 9747 AG Groningen, The Netherlands; Institute for Chemistry and Biology of the Marine Environment, Carl von Ossietzky University Oldenburg, Schleusenstr. 1, 26382 Wilhelmshaven, Germany; Molecular Genetics Group, Groningen Biomolecular Sciences and Biotechnology Institute, University of Groningen, Nijenborgh 7, 9747 AG Groningen, The Netherlands; Groningen Institute for Evolutionary Life Sciences (GELIFES), University of Groningen, Nijenborgh 7, 9747 AG Groningen, The Netherlands

**Keywords:** experimental evolution, adaptive diversification, metabolic trade-offs, metabolic network analysis, central carbon metabolism, *Lactococcus cremoris*

## Abstract

Mitigating trade-offs between different resource-utilization functions is key to an organism’s ecological and evolutionary success. These trade-offs often reflect metabolic constraints with a complex molecular underpinning; therefore, their consequences for evolutionary processes have remained elusive. Here, we investigate how metabolic architecture induces resource-utilization constraints and how these constraints, in turn, elicit evolutionary specialization and diversification. Guided by the metabolic network structure of the bacterium *Lactococcus cremoris*, we selected two carbon sources (fructose and galactose) with predicted coutilization constraints. By evolving *L. cremoris* on either fructose, galactose, or a mix of both sugars, we imposed selection favoring divergent metabolic specializations or coutilization of both resources, respectively. Phenotypic characterization revealed the evolution of either fructose or galactose specialists in the single-sugar treatments. In the mixed-sugar regime, we observed adaptive diversification: both specialists coexisted, and no generalist evolved. Divergence from the ancestral phenotype occurred at key pathway junctions in the central carbon metabolism. Fructose specialists evolved mutations in the *fbp* and *pfk* genes that appear to balance anabolic and catabolic carbon fluxes. Galactose specialists evolved increased expression of *pgmA* (the primary metabolic bottleneck of galactose metabolism) and silencing of *ptnABCD* (the main glucose transporter) and *ldh* (regulator/enzyme of downstream carbon metabolism). Overall, our study shows how metabolic network architecture and historical contingency serve to predict targets of selection and inform the functional interpretation of evolved mutations. The elucidation of the relationship between molecular constraints and phenotypic trade-offs contributes to an integrative understanding of evolutionary specialization and diversification.

## Introduction

Diversity in metabolic strategy is a principal factor underlying variation within and between major groups of organisms (Brown et al. 2004; Schramski et al. 2015 ). Resource-utilization strategies are intimately linked to metabolic constraints. The transport and processing of resources to release energy (catabolism) and the construction of cellular building blocks (anabolism) are subject to physical, biochemical, or thermodynamical laws that constrain both the rate of conversions and the range of feasible resource-utilization strategies. Moreover, many of the feasible metabolic strategies may never evolve in practice, because they require substantial evolutionary innovation (Jaeger et al. 2012). In other words, metabolism is also constrained by historical contingency, reflected by the preexisting pathway architecture and regulatory machinery (Meijer et al. 2020).

**Fig. 1.**
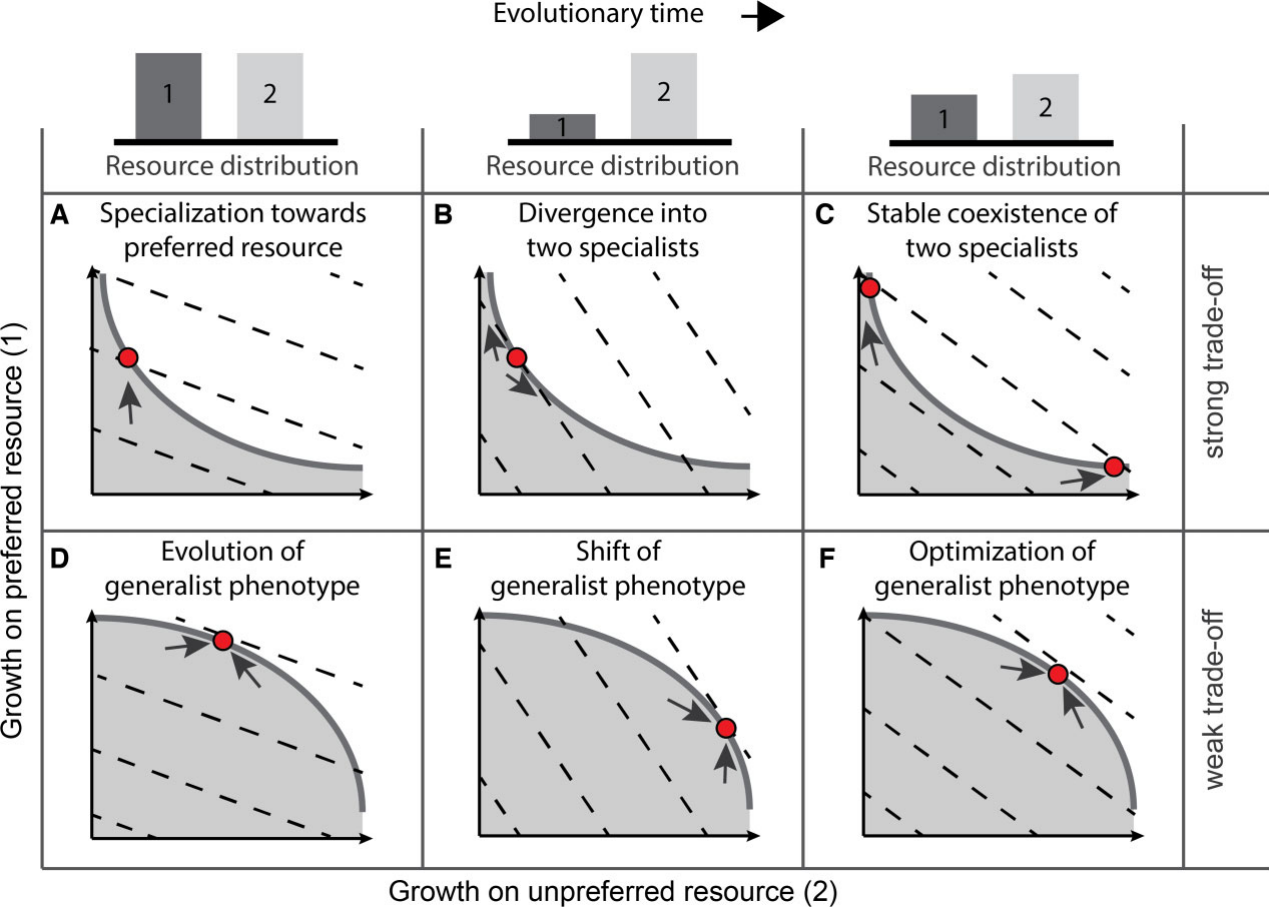
Eco-evolutionary theory of adaptive diversification. Snapshots of the evolutionary trajectories of (sub)populations (represented by filled circles) exposed to strong (*A*–*C*) or weak trade-offs (*D*–*F*) between the utilization of a preferred (1) versus unpreferred (2) resource. Trade-off curves, indicated by solid gray lines, mark the boundary between evolutionarily feasible (shaded area below the curve) and unfeasible (area above the curve) phenotype combinations. Dashed lines represent contours of equal fitness (fitness increases toward the upper right corner of each diagram); note that the shape of the fitness landscape is dependent on the relative proportions between the available resources (as indicated by bar charts above each column). Arrows indicate the direction of adaptation, proceeding in the direction of higher fitness, whereas following the trade-off curve. Bar plots on top show resource distribution (relative availability). (*A*) Strong trade-offs initially lead to specialization on a preferred resource to achieve highest fitness. (*B*) However, the ensuing drop in the concentration of the preferred resource creates conditions favorable for the evolution of a second type specializing on the consumption of the alternative resource 2. (*C*) In the end, the two specialists can attain stable coexistence, maintained by frequency-dependent competition for resources. (*D*) Weak trade-offs allow the evolution of a generalist phenotype, that may subsequently (*E*) respond to consumption-induced resource change, eventually leading to (*F*) a coutilization profile that matches the availability of resources. See Appendix and supplementary fig. S1, Supplementary Material online for further details.

A functional consequence of metabolic constraints is that they can give rise to trade-offs between resource-utilization strategies (Pfeiffer et al. 2001; Novak et al. 2006; Bennett and Lenski 2007; MacLean 2008; Cooper and Lenski 2010; Jasmin et al. 2012). By preventing the efficient coutilization of multiple resources, such constraints may force the organism to compromise on its maximum growth rate when it maintains a generalist resource-utilization strategy. Strong trade-offs may even induce specialization on one alternative resource or another, providing an opportunity for the evolution of a population-level polymorphism of metabolic specialists (fig. 1). The conditions for such diversification have been carefully analyzed in the theoretical literature (summarized in the Appendix, see also supplementary fig. S1, Supplementary Material online). In a nutshell, the theory indicates that diversification relies on resource competition or other ecological mechanisms (Dieckmann and Doebeli 1999; Doebeli and Dieckmann 2000) to create a dynamic (frequency-dependent) regime of selection, which is necessary to maintain the coexistence of multiple specialists (Geritz et al. 1998). Second, it depends on the presence of a sufficiently strong trade-off between alternative resource-utilization strategies, to prevent the evolution of a generalist.

Microbial laboratory evolution experiments, followed by genomic analyses (‘evolve and resequence’), have been widely used to evaluate this theory, and study the evolution of resource specialization and metabolic diversification (Beaumont et al. 2009; Burke et al. 2010; Meyer et al. 2012; Good et al. 2017; Lenski 2017). A remaining challenge in microbial evolution experiments is to explain or predict how metabolic adaptations are constrained, and under what conditions such constraints give rise to evolutionary trade-offs. Although the metabolism of many microorganisms is well resolved in terms of its constituent parts (genes, proteins, and regulatory mechanisms) and their interaction, the functions generally selected for in microbial experimental evolution are growth rate and/or yield (Bachmann et al. 2017), which rely on high-level metabolic functions (e.g., homeostasis, energy allocation, replication, and the synthesis of building blocks) (Pfeiffer et al. 2001; Molenaar et al. 2009; Schuetz et al. 2012). All of these are governed by complex developmental and regulatory networks (Buescher et al. 2012; Bassalo et al. 2018), and constrained on multiple levels by trade-offs (e.g., between rate and yield, anabolism and catabolism, or respiration and fermentation) (Molenaar et al. 2009; Bono et al. 2017; Cheng et al. 2019). Although this intricate network of metabolic and regulatory interactions obviously complicates the relationship between genetic and phenotypic variation, it provides, at the same time, a framework for developing hypotheses on the phenotypic effects of mutations and the molecular basis of adaptation. In particular, combined with knowledge on prevailing environmental conditions in the past, it can serve to predict targets of selection against a background of preexisting biases in resource use, the tuning of the regulatory network owing to prior adaptation and other forms of historical contingency.

### Predicting Resource Coutilization Constraints in the Metabolism of *Lactococcus cremoris*

To explore the potential of this functional network perspective, we evaluate whether knowledge of the metabolic architecture, that is, the way in which metabolic processes are connected through a series of biochemical reactions, can successfully predict how multiple interdependent functions interact agonistically or antagonistically to facilitate or constrain targeted metabolic adaptations. In particular, we investigate the potential to predict target loci for adaptation in an evolutionary diversification experiment, where we selected alternative carbon-utilization strategies in the lactic acid bacterium *L. cremoris*.

Carbon metabolism has a large impact on the rate and yield of microbial growth, because of its central role in energy production and the assimilation of cell material from resources. The central carbon metabolism of the model organism used in our experiment, *L. cremoris* MG1363 (formerly called *Lactococcus lactis ssp cremoris*), has been resolved in detail, owing to its importance in milk product fermentation (Neves et al. 2005; Teusink and Molenaar 2017). Over the course of its domestication to the dairy habitat, *L. cremoris* has been adapted for fast ‘batch mode’ growth on the glucose moiety of lactose (milk sugar) (Bachmann et al. 2012; Kok et al. 2017; Kleerebezem et al. 2020). Dairy strains of *L. cremoris* achieve maximal growth rate on glucose and, when excess glucose is available, catabolite repression downregulates other metabolic pathways in order to maximize growth rate (Zomer et al. 2007). *Lactococcus cremoris* is also able to grow on fructose and galactose as sole carbon source, though the pathways for processing these sugars are subject to catabolite repression if glucose is available (Grossiord et al. 2003; Barrière et al. 2005).

**Fig. 2.**
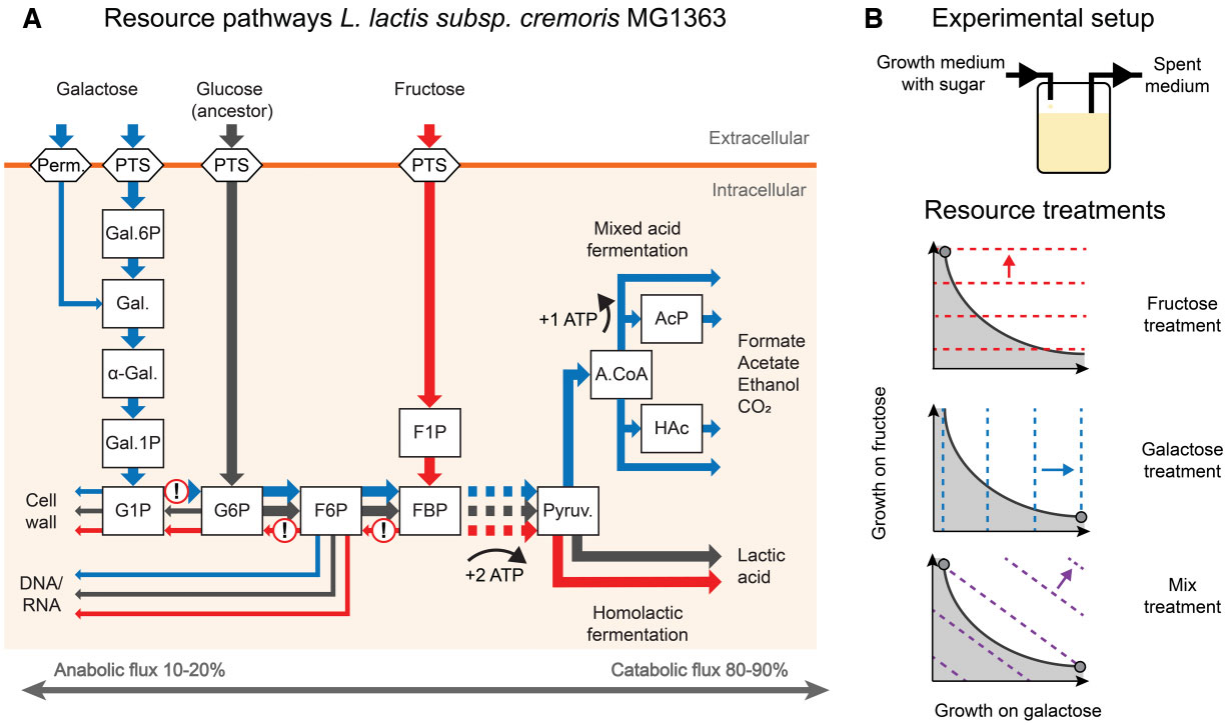
Schematic representation of the metabolic architecture of the central carbon metabolism of *Lactococcus cremoris* MG1363 and experimental setup. (*A*) Metabolites are shown as squares. Metabolic fluxes are visualized by color-coded arrows indicating the net direction of anabolic and catabolic fluxes during growth on glucose (gray), fructose (red), and galactose (blue). Arrow widths are indicative of relative flux rates. Exclamation marks indicate reversals in the direction of net fluxes relative to the ancestral glucose-adapted metabolism. Reversed reactions (from left to right) are coded by pgmA, pgiA, pfk (catabolic direction), and fbp (anabolic direction). Perm., permease; PTS, phosphotransferase system; Gal.6P, galactose-6-phosphate; Gal., galactose; α-Gal., alpha-galactose; Gal.1P, galactose-1-phosphate; G1P, glucose-1-phosphate; G6P, glucose-6-phosphate; F6P, fructose-6-phosphate; F1P, fructose-1-phosphate; FBP, fructose-1-6-biphosphate; Pyruv., pyruvate; A.CoA, acetyl-CoA; HAc, acetaldehyde; AcP, acetyl phosphate. For a more detailed and complete visualization of the glycolytic fluxes see supplementary fig. S5, Supplementary Material online. (*B*) Continuous culture based experimental evolution with fructose and galactose as sole carbon sources. Predicted endpoints of evolution (gray dots) in the three experimental treatments, based on the assumption that fructose and galactose metabolism are subject to a strong trade-off. Dotted lines indicate fitness isoclines and arrows indicate the direction of selection in each treatment. Solid lines indicate the trade-off curve between galactose and fructose growth performance, demarcating the evolutionary feasible phenotype combinations (shaded area).

The fructose and galactose pathways feed into the central carbon metabolism at different entry points, which are located, respectively, downstream and upstream of where anabolic pathways (involved in the synthesis of cell wall components and nucleic acids) branch off from the core glycolytic pathway (fig. 2*A*). Growth on fructose and galactose, therefore, requires a different organization of catabolic versus anabolic fluxes than in the glucose-adapted ancestor, so that the metabolic flux pattern expressed by the ancestral strain is likely suboptimal for growth on both fructose and galactose (fig. 2*A*; supplementary fig. S5, Supplementary Material online).

Previous analyses of glucose-adapted dairy strains support these hypotheses. First, metabolic flux optimization is known to prioritize high catabolic fluxes at the branch points with anabolic pathways, because only a limited carbon flux suffices to supply the anabolic pathways. Catabolic fluxes outweigh anabolic fluxes by a factor of 10–20 in the carbon metabolism of *L. cremoris* (Novák and Loubiere 2000), and this ratio is optimized to maximize the yield and rate of metabolism depending on the concentration of available nutrients (Molenaar et al. 2009). Second, dairy strains of *L. cremoris* exhibit a relatively high growth rate (0.82 h^−1^; supplementary fig. S3, Supplementary Material online) but low growth yield (two ATP per sugar molecule) when growing on fructose. Consistent with the fact that fructose enters in the upper glycolytic pathway at the level of FBP (fructose-1,6-bisphosphate; downstream of the entry-point for glucose), fructose metabolism is constrained by insufficient anabolic flux between FBP and fructose-6-phosphate (F6P) (Looijesteijn et al. 1999); in the reverse direction, the conversion from F6P to FBP is part of the catabolic pathway during growth on glucose. Third, *L. cremoris* growing on galactose as the sole carbon source exhibit high growth yield (three ATP per sugar molecule) but a low growth rate (0.43 h^−1^; supplementary fig. S3, Supplementary Material online). Expression experiments indicated that this slow growth is attributable to a low expression of *pgmA* (Neves et al. 2006, 2010), the enzyme that catalyzes the reversible conversion between glucose-1-phosphate (G1P) and glucose-6-phosphate (G6P). Low *pgmA* expression is sufficient to support the anabolic flux (G6P to G1P) when cells are growing on glucose, but generates a major metabolic bottleneck during growth on galactose (when the G1P to G6P reaction functions as part of the catabolic pathway).

Collectively, these differences suggest that galactose metabolism is a relatively more difficult metabolic specialization to evolve for the glucose-adapted ancestral *L. cremoris*. Moreover, given that the required modifications of the anabolic versus catabolic flux relationships are opposite between fructose and galactose metabolism, we also expect that the growth rates on these sugars are subject to a strong trade-off relative to each other.

### Experimental Design

To test our hypothesis of the metabolic architecture of *L. cremoris*—specifically, the prediction that resource adaptation will target pathway branch points and reactions in glycolysis where the distribution or net direction of metabolic fluxes needs to be adjusted relative to the ancestral (glucose preadapted) state—we imposed selection on carbon utilization in *L. cremoris* in an evolution experiment (fig. 2*B*). To apply a controlled selective pressure and, at the same time, allow frequency-dependent ecological feedback mechanisms to operate, we evolved strains in a customizable chemostat system (Ekkers et al. 2020), rather than in serial dilution cultures, which inherently exhibit strong temporal fluctuations in growth conditions (Gresham and Dunham 2014; Ekkers et al. 2020). The experiment included three treatments, corresponding to alternative selective regimes: two single-sugar treatments with either fructose or galactose supplemented growth media and a third treatment where populations were supplied with an equal mix of fructose and galactose (mix treatment). The single-sugar treatments are expected to elicit divergent adaptations to, respectively, fructose and galactose utilization (fig. 2*B*). The mix treatment is predicted to yield either a polymorphism of metabolic specialists (or a single-strain specialized on one of the two resources), if the trade-off between fructose and galactose metabolism is strong (figs. 1*A–C* and 2*B*; Appendix; Rueffler et al. 2006; Herron and Doebeli 2013) or, if the trade-off is weak, the evolution of a generalist that can coutilize both resources and optimally tune the consumption of both sugars to their relative availability in the growth medium (fig. 1*D–F*; Appendix). We then characterize the adaptive mutations that evolved in divergently selected lines and interpret their function in the context of the metabolic network (see supplementary fig. S2, Supplementary Material online for an overview of the Methods).

## Results

### Phenotypic Adaptation Leads to Divergence of Two Specialist Phenotypes

**Fig. 3.**
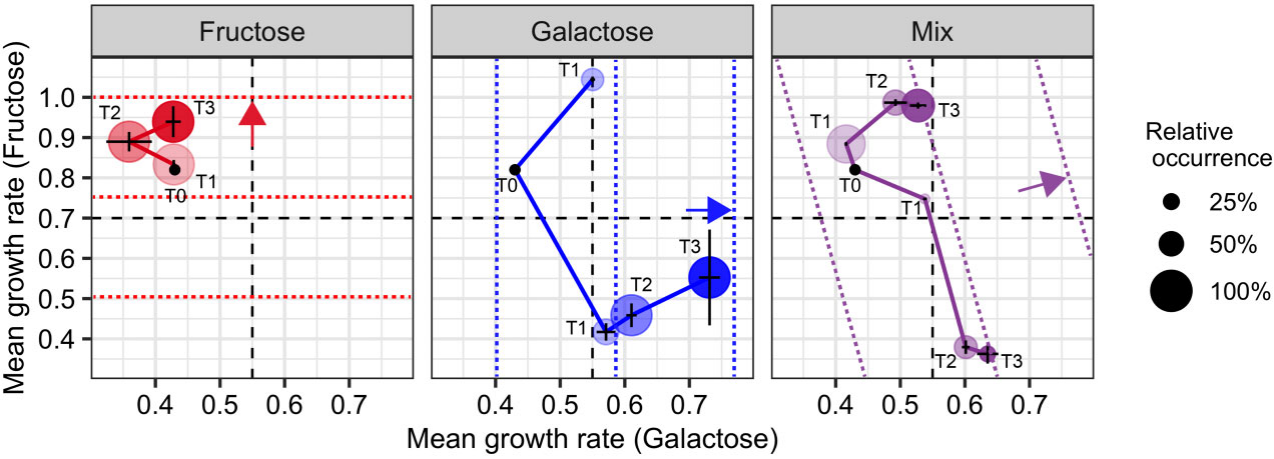
Evolutionary trajectories. Evolutionary phenotypic trajectories of fructose (left), galactose (middle), and mix (right) treatments, quantified by the max growth rates on fructose and galactose supplemented CDMPC medium. Data points are calculated based on growth rate measurements of single strains/genotypes sampled from the different populations across three timepoints (T1, T2, and T3; point T0 represents the ancestral strain). Because of growth rate differences between treatments the length of the experiment in number of generations was unequal between treatments, with the total duration of the fructose, galactose, and mix-treatment amounting to, respectively, 1111, 565, and 575 generations. Therefore, timepoints T1, T2, and T3 were selected per treatment, such that they were equally spread over the growth rate performance increase achieved throughout the experiment (supplementary fig. S6, Supplementary Material online). Growth rate data were clustered to distinguish phenotypic groups across replicates. The sizes of the colored circles are indicative of the frequency of each group of phenotypes, and error bars indicate SD within each cluster. Colored short-dashed lines and arrows give an indication of the contours of the fitness landscape and the direction of selection in each treatment, as in figure 2; long-dashed lines cross through the midpoint of the interval of group-average growth-rate values that we observed in our dataset (*F* = 0.703; *G* = 0.545) and roughly demarcate regions of phenotype space corresponding to different strategies: left upper corner (FS, right bottom corner GS, right top corner, and left bottom corner [generalists]).

Phenotypic analysis of the evolved strains showed that the growth-rate improvements in the fructose and galactose treatments were associated with specialization on the available sugar (fig. 3). The evolutionary trajectories reconstructed from the phenotypic data revealed that the two specialist phenotypes, the fructose specialist (FS) and the galactose specialist (GS), continued to diverge as the evolution experiment progressed, indicating that the evolved strains were continuously selected for improved performance on the selected sugar, although the rate of improvement leveled off halfway through the experiment (fig. 3). Unless stated otherwise, FS and GS from the different treatments refer to phenotypic clusters of strains from four independent replicates that evolved in parallel (supplementary fig. S9, Supplementary Material online).

In the mix treatment, we observed the evolution of a phenotypic polymorphism, consisting of a FS^mix^ coexisting with a GS^mix^ (fig. 3; throughout, superscripts refer to the experimental treatment—fructose, galactose or mix—from which the strains were sampled). These coexisting phenotypes diverged through time, following similar trajectories as the FS^fru^ and GS^gal^ from the single-sugar treatments. Each of the replicate populations from the mix treatment contained strains occurring in both galactose and FS clusters (supplementary fig. S10, Supplementary Material online), indicating that the divergence of the ancestor into two specialists occurred in parallel across all four replicates of this treatment.

With regard to the phenotypic growth-rate characterizations, the FS remained closer to the ancestral phenotype than the GS (fig. 3). This observation is consistent with the asymmetry in the relative growth improvement observed during the running of the experiment (supplementary fig. S6, Supplementary Material online), which was lower in the fructose treatment than in the galactose treatment. In fact, when the ancestral phenotype was included in the phenotypic analysis it always clustered with the FS (data not shown).

### Specialist Phenotypes Display Consistent Trade-Off Patterns

The growth improvement of the GS (GS^gal^ and GS^mix^) was generally associated with decreased performance on fructose compared with the ancestor (i.e., in phenotypic assays performed on strains sampled from the evolution experiment, fig. 3; supplementary fig. S8, Supplementary Material online). In the galactose treatment, the presence of two clusters at T1 indicates that initial improvement of galactose metabolism can occur either with or without trading off on fructose performance; however, additional improvement in growth on galactose only evolved in concert with a trade-off on fructose performance (single clusters at T2 and T3). FS^fru^ and FS^mix^, however, showed no trade-off when they were cultured on galactose (fig. 3; supplementary fig. S8, Supplementary Material online), which may reflect the initial poor performance of the ancestral strain on galactose relative to its growth rate on fructose. Nevertheless, a clear trade-off pattern (negative relation) between the evolved FS and GS emerged both in the mix treatment as well as between the independently evolved populations observed in the isolated fructose and galactose treatments (supplementary fig. S9, Supplementary Material online). Importantly, the failure to observe the evolution of a generalist in the mix-treatment supports the notion that there is a strong performance trade-off between fructose and galactose metabolic adaptation (Appendix). Instead of a generalist, we found a polymorphism of FS and GS in all replicates of the mix treatment (fig. 3; supplementary fig. S10, Supplementary Material online), even though the evolving populations had access to both sugars and selection promotes the evolution of a generalist by favoring a simultaneous performance increase on fructose and galactose.

Toward the end of the experiment (T3), we observed the emergence of a phenotype that exhibited high growth rate on both galactose and fructose in one replicate of the galactose treatment (supplementary fig. S7, Supplementary Material online). Although this rare phenotype (hereafter denoted as GS*^gal^) appears to be a generalist, it evolved only once (in one replicate) and in a treatment where improvement on fructose was not selected for (i.e., the galactose-only treatment). Moreover, GS*^gal^ did not evolve directly from the ancestral strain, it only evolved secondarily from a monomorphic GS population (T2). Surprisingly, such a generalist strategy did not evolve in the mix treatment where high performance on galactose and fructose was favored by natural selection.

We interpreted the evolution of GS*^gal^ as an indication that trade-offs can change as phenotypes continue to evolve in response to selection for alternative resource utilization. To test this hypothesis, we selected a subset of representative specialist strains from each treatment and evaluated their growth rate and yield on three additional carbon sources (glucose, mannose, or trehalose). Glucose was the preferred sugar for the ancestral strain, which was preadapted for efficient glucose utilization; the other two sugars were selected because of the large metabolic overlap between the galactose and trehalose pathways, and between the glucose and mannose pathways, respectively. Fructose does not share overlap in peripheral metabolic pathways with other monosaccharides.

**Fig. 4.**
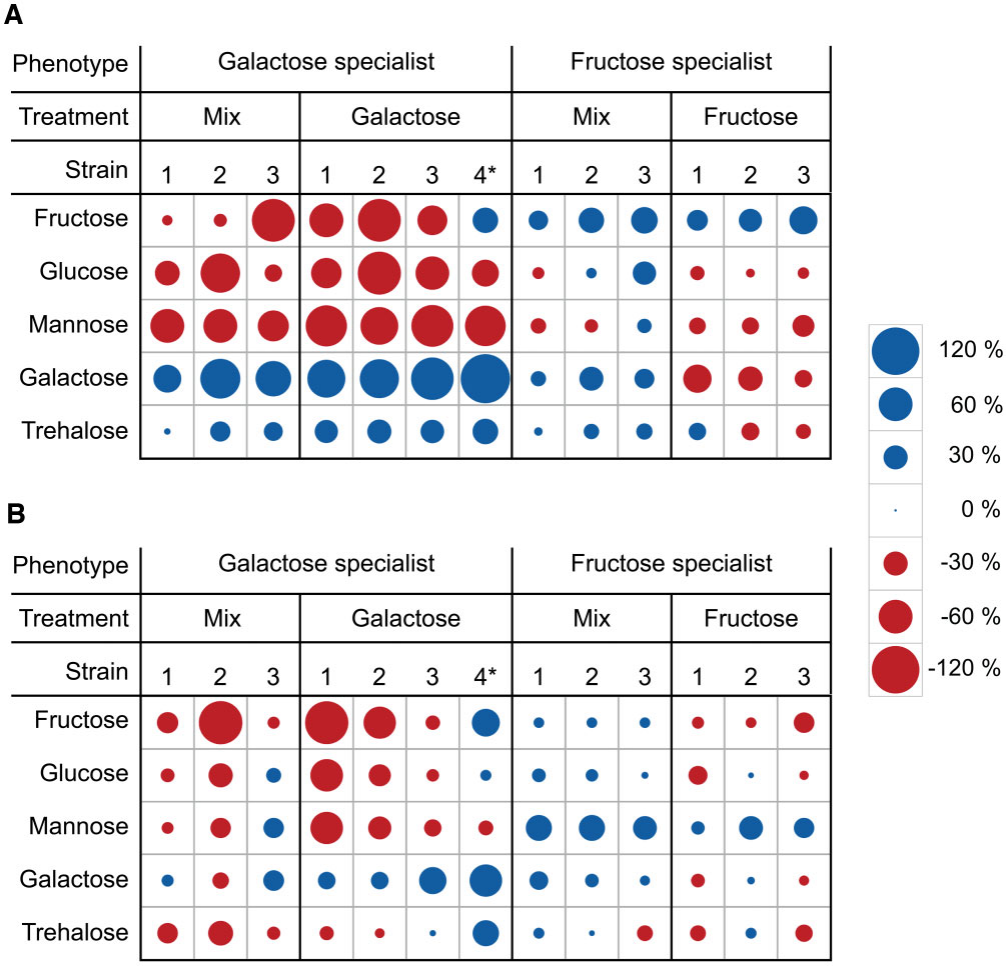
Metabolic performance profile of selected FS and GS strains. The evolved strains show different abilities to utilize metabolic resources. The strains were grown in triplicates in batch on fructose, glucose, mannose, galactose, or trehalose (1% wt/v) supplemented CDMPC. Circle size is proportional to the magnitude of the relative increase (blue) or decrease (red) in (*A*) maximum growth rate or (*B*) biomass yield compared with the ancestral strain. See supplementary fig. S3, Supplementary Material online for absolute values. The * on the GS^gal^ strain number 4 refers to the GS^*gal^ strain. The names of the selected strains in the supplementary table S1, Supplementary Material online are: for the GS, mix-treatment: strain 1 (M4_t3_fg), strain 2 (M4_t3_eg), strain 3 (M2_t3_ef). GS Galactose treatment: strain 1 (G1_t3_ag), strain 2 (G2_t3_eg), strain 3 (G2_t3_fg), strain 4* (G2_t3_cg). FS, mix-treatment: strain 1 (M4_t3_ef), strain 2 (M2_t3_bf), strain 3 (M4_t3_ff). FS fructose treatment: strain 1 (F4_t3_df), strain 2 (F3_t3_ff), strain 3 (F3_t3_af).

This experiment revealed that the growth-rate trade-offs detected between fructose and galactose utilization in specialist phenotypes also extended to other carbon sources (fig. 4*A*; supplementary figs. S3 and S4, Supplementary Material online). When compared with the ancestor, strains with GS^gal^ and GS^mix^ phenotypes display a lower growth rate on glucose, mannose and, to a lesser extent, fructose. Conversely, the FS^fru^ and FS^mix^ strains showed similar, or slightly reduced, growth rates on glucose and mannose when compared with the ancestor. An opposite, but overall less consistent pattern was observed for growth on trehalose: relative to the ancestor, GS strains exhibited an increased growth rate on this sugar, whereas FS strains maintained a similar growth rate as the ancestral strain. For growth yield, no consistent pattern was found for GS strains, and FS strains consistently exhibited a high yield for mannose (fig. 4*B*; supplementary figs. S3 and S4, Supplementary Material online). The strain from the rare GS*^gal^ phenotype showed similar trade-off patterns as the other GS strains, except for its improved growth on fructose and overall higher yield on all sugars except for mannose. Overall, the GS strains deviated considerably in their performance on a variety of carbon sources, whereas the FS strains retained a resource-utilization pattern similar to the ancestor.

### Genetic Analysis Identifies Targets of Selection

To identify the molecular changes underlying the observed phenotypic changes in resource utilization, we performed whole-genome sequencing on 48 single strains and 36 population metagenomic samples (supplementary figs. S2, S12, and S13, Supplementary Material online). Based on the single-strain analysis, we identified a total of five annotated loci that most strongly correlated with resource specialization (see Materials and Methods for the criteria used to identify these mutations and supplementary fig. S12*A–D*, Supplementary Material online), namely *pgmA*, *ptnABCD*, and *ldh* for GS and *fbp* and *pfk* for FS. The population analysis, combined with the single-strain analysis, revealed that mutations in the selected genes evolved independently in multiple replicate populations and treatments (supplementary fig. S12*E* and *F*, Supplementary Material online). Frequencies and total occurrence of selected genes were generally lower in poorly performing replicates. For a functional analysis of the evolved mutations, we selected five strains (one strain from each characteristic phenotype of each treatment at T3) and performed expression and enzymatic analyses; the combinations of mutations present in the selected strains, as well as the strains names, are listed in supplementary fig. S12*G*, Supplementary Material online.

### FS Evolve to Rebalance Anabolic and Catabolic Flux

**Fig. 5.**
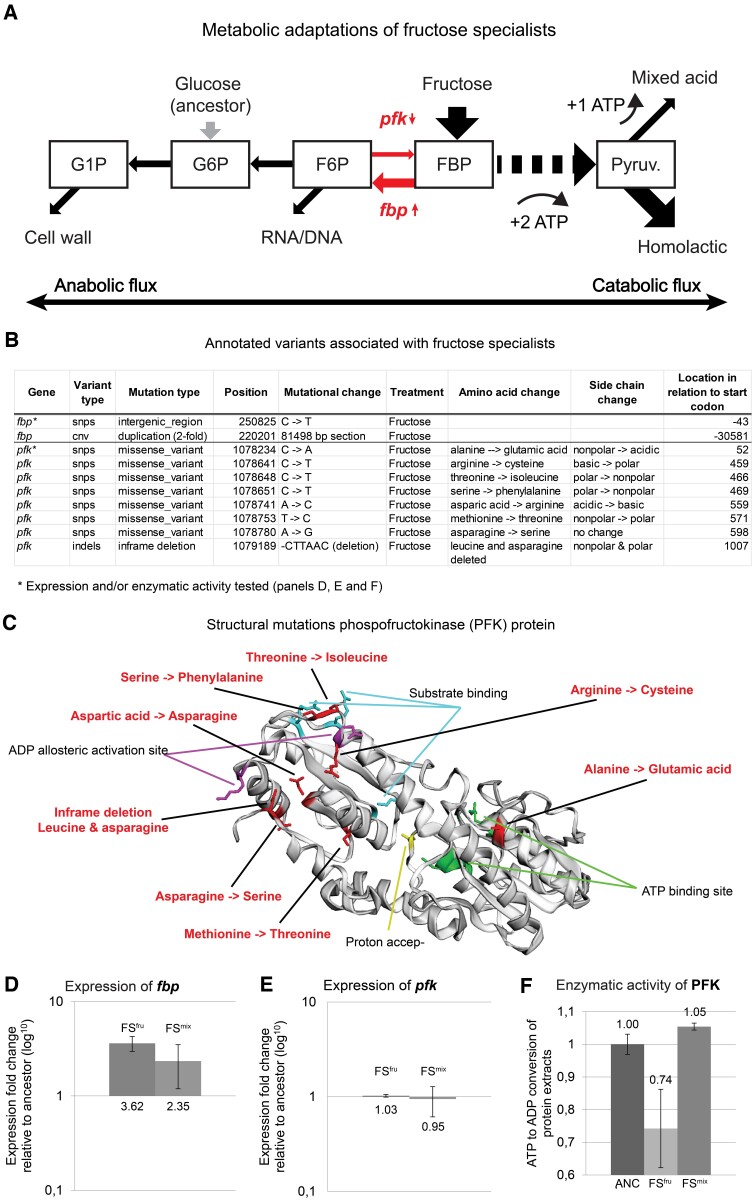
Analysis of *loci* associated with fructose specialization. (*A*) Mutated genes (red) in the FS mapped to the metabolic architecture of upper glycolysis. Arrows indicate increase (upward pointing) or decrease (downward pointing) in activity. Metabolites are shown as squares. The metabolic fluxes are visualized by big arrows indicating the net direction of metabolic flux when metabolizing fructose. Big red arrows indicate mutated metabolic steps. (*B*) Variants of FS associated annotated loci. (*C*) 3D structure of PFK with mutations (in red) and functional sites (in black) annotated. Mutated regions (red), allosteric activation sites (purple), substrate binding sites (blue), magnesium catalytic site and proton acceptor (orange), nucleotide and ATP binding site (green). (*D*) Expression of *fbp* when grown on F-CDMPC, error bars indicate standard deviations (SD). (*E*) Expression of *pfk* when grown on F-CDMPC, error bars indicate SD. (*F*) Enzymatic activity of PFK protein extracts by measuring phosphorylation of fructose-6-phosphate to fructose 1,6-bisphosphate, error bars indicate SD. The names of the selected strains in panels *D*, *E*, *F* correspond to the strain names in the supplementary table S1, Supplementary Material online: ANC, FS^fru^ (F3_t3_ff), FS^mix^ (M4_t3_ff).

The combined population and single-strain genetic analyses showed that FS^fru^ evolved mutations in *fbp* and *pfk*. These genes code for the enzymes that catalyze the reaction FBP → F6P and its reverse reaction F6P → FBP, respectively (fig. 5*A and B*).

*Fbp*—In our experiment, mutations in *fbp* spread in the fructose control treatment at timepoint T1, T2, and T3. *Fbp* codes for FBPase (fructose bisphosphatase), which catalyzes the conversion of FBP to F6P. *Fbp* expression was significantly increased 3.62-fold in the *fbp* intergenic FS^fru^ mutant and 2.35-fold in the FS^mix^ strain without a mutation compared with the ancestral expression levels (fig. 5*D*). The expression data suggest that the FS^fru^ and FS^mix^ evolved an increased anabolic flux from FBP to F6P. This result aligns with earlier work where *fbp* expression was shown to limit both growth rate and yield when *L. cremoris* is exclusively grown on fructose (Looijesteijn et al. 1999).

*Pfk*—Eight different variants of *pfk* evolved independently in FS^fru^ at timepoints T2 and T3, and all mutations occurred in the structural part of the *pfk* gene (fig. 5*A* and *B*). *Pfk* codes for PFK (phosphofructokinase) and is regulated by allosteric inhibition and activation by ATP and ADP, respectively (Papagianni et al. 2007). This regulation allows PFK to mediate the rate of glycolysis in response to the energetic state of the cell through ADP and ATP levels. 3D modeling of the *pfk* protein revealed that structural mutations reside close to the ADP and ATP binding sites; one mutation occurred in the ADP allosteric activation site (fig. 5*C*). Based on these results, we compared the *pfk* expression of evolved FSs growing on fructose. We found that expression of the *pfk* mutant with a mutation in proximity of ATP allosteric site was equal to that of the ancestral strain (fig. 5*E*). However, the *pfk* mutant showed a decrease in enzymatic activity of 25% compared with the ancestor (fig. 5*F*).

We found that both *fbp* and *pfk* mutations occur in the same strain, but we also found strains with a mutation in only one of these genes, indicating that these two mutations can be beneficial independently but also in combination. We speculate that the mutations in *pfk* and *fbp* in the FSs decrease catabolic and increase anabolic activity, respectively, both resulting in an increased net carbon flux in the anabolic direction.

### GS Evolve to Resolve a Metabolic Bottleneck, Tune Sugar Transport, and Modulate Downstream Metabolism

**Fig. 6.**
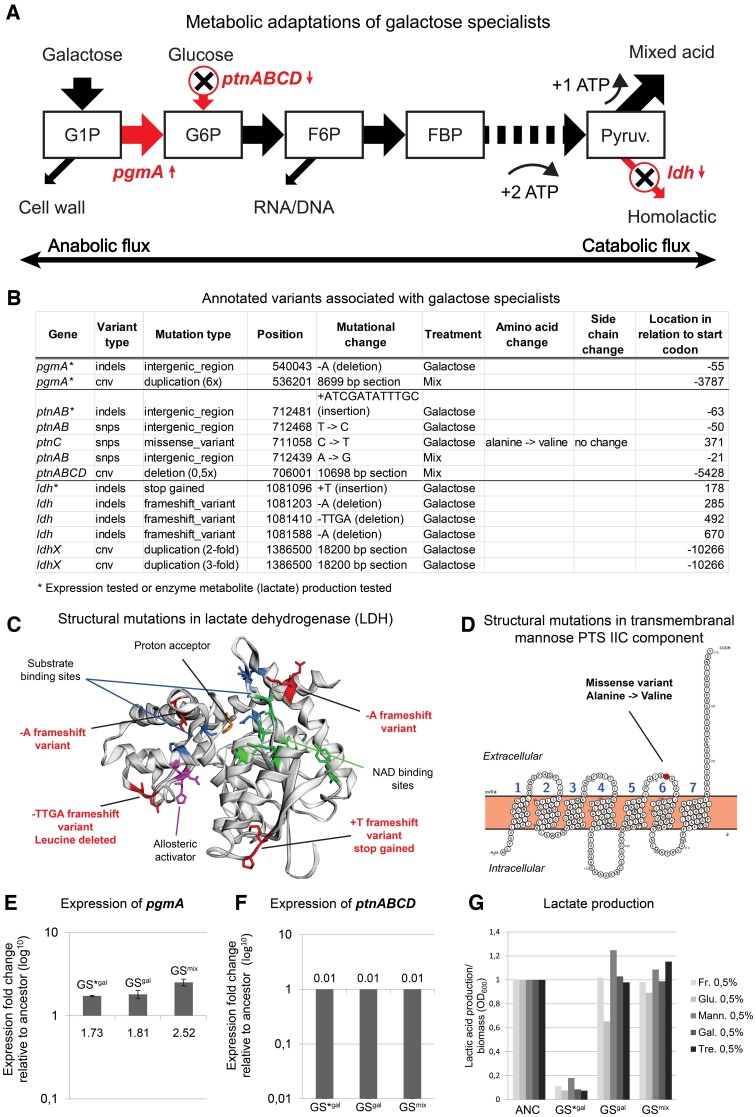
Analysis of *loci* associated with galactose specialization. (*A*) Mutated genes (red) in the GS mapped to the metabolic architecture of upper glycolysis. Small red arrows indicate increase (upward pointing) or decrease (downward pointing) in activity of mutated genes as compared with the ancestral strain. Metabolites are shown as squares. The metabolic fluxes are visualized by big arrows indicating the net direction of metabolic flux when metabolizing galactose. Big red arrows indicate mutated metabolic steps. (*B*) Variants of GS-associated annotated loci. (*C*) 3D structure of LDH with mutations and functional sites annotated. Mutated regions (red), allosteric activation sites (purple), substrate binding sites (blue), proton acceptor (orange), NAD binding site (green). (*D*) Location of structural mutations mapped to protein structure of mannose PTS. (*E*) Expression of pgmA when grown on G-CDMPC, error bars represent SD. (*F*) Expression of ptnABCD when grown on G-CDMPC, error bars represent SD. (*G*) Lactic acid production per unit of biomass on sugar supplemented CDMPC. Optical densities are normalized so that the ancestor (ANC) has OD_600_ equal one. The names of the selected strains in panels E, F, G correspond to the strain names in the supplementary table S1, Supplementary Material online: ANC, GS*^gal^ (G2_t3_cg), GS^gal^ (G2_t3_fg), GS^mix^ (M4_t3_eg).

The combined population and single-strain genetic analyses showed that GS feature mutations in the genes *pgmA*, *ptnABCD*, and *ldh*(*x*) (fig. 6*A*). Most of the variants resided in intergenic regions of the genes or were copy-number variants (CNVs) (fig. 6*B*). Four structural variants were detected in *ldh* (fig. 6*C*) and one in *ptnABCD* (fig. 6*D*).

*PgmA*—The gene *pgmA* codes for α-PGM (α-phosphoglucomutase), which catalyzes the reversible conversion of G1P to G6P. Its activity in *L. cremoris* is essential for the catabolism of galactose using the Leloir pathway in the G1P to G6P direction (fig. 2*A*; Grossiord et al. 1998). It has been experimentally shown that α-PGM is the rate-limiting step of galactose metabolism in *L. cremoris* (Neves et al. 2006, 2010). All the sampled GS^gal^ in timepoint T2 (two strains) and T3 (three strains, including GS*^gal^) featured a mutation in the intergenic region upstream of the *pgmA* gene (fig. 6*B*). The mutation was not found in GS^mix^, which instead evolved a 6-fold duplication of the chromosome region containing the *pgmA* gene (fig. 6*B*). RT-qPCR expression experiments of *pgmA* revealed that expression increased 2-fold in both strains (GS^gal^ and GS*^gal^, fig. 6*E*). The mutation of *pgmA* in GS^mix^ resulted in a 3-fold expression increase (fig. 6*E*). Previous experiments have shown that a plasmid-based 3.8-fold overexpression of *pgmA* in a Δ*pgmA* strain boosted α-PGM activity, increasing galactose consumption by 19% (Neves et al. 2006). Similarly, a 6-fold activity increase in another experiment yielded 25% consumption increase on galactose CDM (Neves et al. 2010). We therefore speculate that the observed increase in *pgmA* expression evolved to resolve the metabolic bottleneck located between G1P and G6P.

*PtnABCD*—The PTS^man^ (mannose phosphotransferase) is coded by *ptnABCD* and was mutated in GS^gal^ and GS^mix^. PTS^man^ is the primary transporter of glucose and mannose metabolism in *L. cremoris* (Castro et al. 2009). Mutations occurred in the intergenic region upstream of the *ptnAB* intracellular components and in the membrane-embedded permease component (*ptnC*) as a structural mutation in the transmembrane section of the protein (fig. 6*D*). Expression analysis of the *ptnABCD* mutant strains from GS^gal^ (including strain GS*^gal^) showed a full inhibition (≤99%) of the expression of *ptnABCD*, indicating that the mutation suppresses the activity of *ptnABCD* (fig. 6*F*). GS^mix^ without a mutation in *ptnABCD* (derived from a population that did contain *ptnABCD* mutants) also displayed full inhibition of *ptnABCD* (fig. 6*F*). This indicates that galactose transport in the GS does not occur via the PTS^man^. The complete silencing of PTS^man^ in both strains suggests that its expression inhibits fast growth on galactose. This result is consistent with a study where mutants of *L. cremoris* that evolved a reduced expression of PTS^man^ display decreased growth on glucose, but enhanced growth on galactose (Kjos et al. 2011).

*Ldh*—The gene *ldh* was mutated in two replicates of GS^gal^, but not in GS^mix^ (fig. 6*B*). Single-strain genetic analysis found *ldh* mutation only in one strain, the GS*^gal^. LDH catalyzes the reversible reduction of pyruvate to lactate with concomitant conversion of NADH to NAD^+^ (fig. 6*A*). This reaction serves to balance redox potential via oxidation (Neves et al. 2005). Four structural *ldh* variants were observed in all GS^gal^ (fig. 6*B* and *C*). All four were frame-shift mutations; one resulted in the gain of a stop codon which occurred in the GS*^gal^ strain, the other three mutations occurred in the proximity of the LDH protein domains that are associated with substrate binding, allosteric activation and NAD^+^ binding (fig. 6*C*). Besides these structural mutations, we also found a large 2- and 3-fold duplicated section that contained *ldhX* (a lactate dehydrogenase homolog). Metabolic analysis of a *ldh* mutant showed a strongly reduced production of lactic acid when grown on a variety of carbon sources (fig. 6*G*) combined with a high growth yield (fig. 4*B*). We conclude that *ldh* mutations have a deleterious effect on lactic acid fermentation and appear to shift the phenotype toward increased mixed-acid fermentation.

## Discussion

### Metabolic Architecture Predicts Trade-Offs that Elicit Specialization and Diversification

Our experimental results provide a striking example of how evolutionary adaptation and divergence are shaped by constraints connected to the architecture of a metabolic network. By considering the requirement of maintaining adequate balance between anabolic and catabolic fluxes, we were able to predict the evolvability of alternative resource-utilization strategies (fructose and galactose specialization) for an ancestral strain preadapted for growth on glucose. Here, we treated network topology (i.e., the way in which metabolic pathways are connected) as a ‘hard’ constraint that imposed restrictions on the range of feasible phenotypes that could potentially evolve over the course of the experiment. Additionally, we considered the control of metabolic fluxes (i.e., set-points and regulatory feedbacks determining pathway activities) as evolvable ‘soft’ constraints that reflected the result of past selection for optimized glucose metabolism and that biased the rate at which alternative specializations could evolve.

Data from our experiment indicate that both types of constraints shape the outcome of evolution. First, *L. cremoris* appears to be able to resolve the soft constraints related to the unique bottlenecks of fructose and galactose metabolism, but at markedly different rates, consistent with the expectation that fructose utilization requires a smaller adjustment of the glucose preadapted ancestral state than galactose utilization. Second, we hypothesize that metabolic network topology acted as a hard constraint preventing efficient simultaneous utilization of fructose and galactose, and the resulting trade-off induced the evolution of a population-level polymorphism in resource specialization in an environment where both sugars were available simultaneously. Taken together, our data exemplify that careful consideration of the constraints and trade-offs that are exposed under a specific environmental-selection regime provides a useful framework to predict, interpret and link phenotypic and genetic evolutionary change. Further validation of the proposed mechanisms can be obtained from in-depth metabolomics measurements to verify that the observed mutations indeed resolve rate-limiting steps in fructose and galactose metabolism.

Future work will also benefit from a better understanding of which metabolic network properties most generically predict constraints. To address this question, similar analyses need to be done for other systems. Opposite net flux directions between alternative specializations have been associated with phenotypic divergence in other experimental evolution studies as well. For instance, divergence between fast and slow switchers in sequential batch culture of *Escherichia coli* growing on a mixture of glucose and acetate, has been explained based on the fact that the ability to switch rapidly from glucose to acetate consumption requires an upstream flux from acetate toward central glycolysis, whereas efficient growth on glucose favors a maximum downstream flux capacity to enable rapid excretion of acetate as a metabolic waste product (Le Gac et al. 2008). Trade-offs have also been associated with the presence of energy-consuming metabolic cycles (van Heerden et al. 2014), or the build-up of toxic metabolic intermediates (Pfeiffer and Bonhoeffer, 2004). The integration of metabolic modeling with experimental data on mutant strains provides a promising approach to identify further structural network properties associated with evolutionary constraints, yet this approach is currently restricted to model species for which kinetic models and metabolomic data for a broad set of mutants are available.

### Specialist Phenotypes Appear to Resolve Metabolic Bottlenecks Related to Preadaptation to Glucose

Divergent evolutionary specializations on fructose and galactose were associated with mutations in key metabolic junctions and transporters (e.g., *fbp*, *pfk*, *ptnABCD*, *pgmA*, and *ldh*). These mutations were located at positions in the metabolic map that were expected to undergo directional (*fbp*, *pfk*, and *pgmA*) or quantitative (*ptnABCD* and *ldh*) changes in metabolic fluxes during the adaptation from glucose to fructose or galactose utilization (fig. 2*A*). The transcriptional and enzymatic changes induced by these key mutations were consistent with the predicted directionality of selection (figs. 5 and 6) inferred from our analysis of anabolic versus catabolic flux bottlenecks in the metabolic network. Galactose specialization was also associated with mutations in *ptnABCD* and *ldh* that we did not predict by our analysis of metabolic bottlenecks. In the ancestor, both *ptnABCD* and *ldh* display a quantitative shift in their expression and activity between glucose and galactose metabolism (Neves et al. 2010; Solopova et al. 2018). A previous study indicated that galactose metabolism was enhanced by inhibitory mutations in the main glucose transporter PTS^man^ (Kjos et al. 2011). Consistent with these findings, PTS^man^ and LDH were found to be inhibited in the galactose adapted strains from our experiment.

### Phenotypic Convergence of FS^mix^ Despite Lack of Genetic Convergence

Notwithstanding the overall pattern of mutations associated with fructose specialization, it is interesting that mutations at the predicted key metabolic junctions were not present in FS^mix^. Still, when the expression of *fbp* was measured in FS^mix^, we find similar expression patterns as in FS^fru^ (fig. 5C) indicating that other currently unidentified variants likely converge to similar transcriptomic adaptations. This assumption is supported by the absence of mutations in the identified target genes in a subset of FS^fru^ strains, despite parallel phenotypic evolution across replicates with and without mutations in these target genes. A follow-up in-depth transcriptomic analysis of the unannotated phenotype-specific loci could potentially reveal which other variants cause these phenotypic and transcriptomic characteristics. Additionally, our analysis may have missed adaptive variants that occur only once or twice in single loci, which were filtered out as a consequence of the application of strict criteria for identifying candidate loci (see Materials and Methods). Finally, it is possible that selection on fructose utilization has been weaker in the mix treatment, especially if the constraints originating at *pfk-fbp* are exposed only during selection at higher metabolic rates. Such conditions have potentially occurred less frequently in the mix treatment, which had generally lower fructose availability (50% less) and lower dilution rates (supplementary fig. S6, Supplementary Material online).

An alternative explanation for the apparent lack of mutations in the mix treatment relates to the ecological conditions induced in the mix treatment, and the fact that they allow for cometabolization of fructose with galactose, which would potentially be an alternative way to resolve the anabolic bottleneck. Phenotypic analysis of FS^mix^ strains showed that they improved their growth rate on galactose toward the end of the experiment, whereas this was not observed for the FSs from the fructose treatment (fig. 3). The FSs isolated from the mix treatment also did not decline in metabolic performance on other sugars, as was the case for the FSs from the fructose treatment (fig. 4*A*). Taken together, this pattern could indicate that the FSs from the mix treatment adapted to cometabolize galactose to supply anabolic fluxes. This principle was demonstrated earlier for the cometabolization of glucose, where *fbp* limitation of fructose-grown *L. lactis* cells could be resolved by the addition of a small amount of glucose to the medium (Looijesteijn et al. 1999). Because the improvement on galactose was relatively small, FSs from the mix treatment were still far from classifying as a generalist phenotype. In fact, GSs *coevolved* in all replicates of the mix treatment where FSs appeared to evolve cometabolization of galactose.

### Rate of Molecular Evolution

In our sequence analysis, we considered not only single nucleotide polymorphisms (SNPs) and insertions and deletions (indels) but also performed CNV analysis (see Materials and Methods). CNVs comprised most of the observed mutations (89%; supplementary fig. S13, Supplementary Material online), which were either large deletions (19%) or duplications (70%); SNPs and indels only made up 11% of total mutations. The consideration of CNVs in our study resulted in an average amount of mutations considerably higher than the amount found recently in a similar evolution experiment performed with *L. lactis* where CNVs were not analyzed (Price et al., 2019). An additional potential explanation for the observed higher amount of mutations is that we evolved *L. lactis* MG1363 on sugars that it was not preadapted to (galactose and/or fructose), whereas in Price et al. (2019) glucose was the primary carbon source. Thus, our experiment likely provided a larger scope for growth-rate optimization and/or a larger target for adaptive mutations, whereas less mutations were required to evolve toward the local fitness optimum in the experiment of Price et al. (2019). Lastly, unlike Price et al. (2019), we dynamically increased the dilution of the bioreactors in order to maintain constant population density and continually select for higher growth rate (keeping selection force constant throughout the evolution experiment). Such unidirectional selection regime could substantially speed up adaptive evolution by more effectively selecting for newly emerging adaptive variants (faster growers) within the population and, thus, fixating beneficial mutations at a higher rate, resulting in faster evolutionary change and more genetic variants (Ekkers et al. 2020; Gresham and Dunham 2014).

### The Evolved GS*^gal^ Strain Exhibits Unexpected Growth Characteristics with Potential Relevance for Industry

Generally, *L. lactis* only approaches high growth rates under homolactic fermentation, which is crucially dependent on LDH activity. The GS*^gal^ strain had a loss-of-function *ldh* mutation (fig. 6*G*), but showed the highest growth rate on galactose and, surprisingly, a high increase in growth on fructose together with low growth on glucose and mannose (fig. 4*A*). During our experiments, we noticed that overnight cultures of the evolved GS*^gal^ strain produce a strong diacetyl aroma; yet, we did not follow-up on this observation with quantitative analysis to confirm the increased activity of the acetoin branch of the downstream metabolism. Performing such an experiment would be worthwhile to explore potential applications in the dairy industry, where a strain with the unusual combination of a high growth rate on galactose, low growth rate on glucose, and mixed-acid mode of growth could find potential use as a galactose-scavenger (Neves et al. 2010).

### Specialization has Pleiotropic Effects on Growth on other Carbon Sources, Correlated to Metabolic Pathway Overlap

By quantifying the growth rate and yield of evolved FS and GS on a range of other sugars (namely, glucose, mannose, and trehalose), we observed that evolutionary specialization generalizes to a broader pattern of resource-utilization trade-offs. In order to explore whether the observed pleiotropic effects are predictable from the metabolic network architecture, we mapped the observed metabolic adaptations of FS and GS onto the hardwired glycolytic backbone (supplementary fig. S14, Supplementary Material online), and attempted to infer their consequences for the growth on a broader range of carbon sources, considering flux directionality constraints and the basic requirement of maintaining appropriate relationships between catabolic and anabolic fluxes based on information from metabolic databases and literature. FS^fru^ that exhibit mutations in *fbp* and *pfk* display a pattern of negative growth effects on all other resources (glucose, mannose, galactose, and trehalose) (fig. 4*A*). This pattern is consistent with the unique low entry-point of fructose in glycolysis compared with the other sugars (supplementary fig. S14, Supplementary Material online). Based on previous research, it is expected that reduced PFK activity will negatively impact growth on other sugars that enter further upstream. Indeed, a 2-fold decrease in PFK activity in *L. lactis* can lead to 57–70% decrease in growth rate on glucose (Andersen et al. 2020). The metabolic profile of GS shows a pattern of both positive (trehalose) and negative (fructose, glucose, and mannose) growth effects (fig. 4*A*). The observed pattern of pleiotropy is consistent with the predicted flux constraints based on their pathway topology (supplementary fig. S14, Supplementary Material online). The disaccharide trehalose connects to glycolysis at the G1P and G6P junctions with an equal moiety (Andersson et al. 2001), so the net flux direction leads downstream from G1P to G6P, as is the case for galactose metabolism. The increased flux between G1P and G6P resulting from *pgmA* overexpression could therefore also benefit trehalose metabolism (supplementary fig. S14, Supplementary Material online). Moreover, trehalose and galactose metabolism exhibits similar regulatory and metabolic responses (Qian et al. 1997). The observed decreased growth on fructose, glucose, and mannose in GS is not surprising because of inhibition of the PTS^man^ and LDH. PTS^man^ is the primary sugar import system for both glucose and mannose and *L. cremoris* achieves its highest growth rate by adopting a homolactic growth mode on both sugars, which is dependent on high LDH activity. The fact that fructose is also partially transported through PTS^man^ (Benthin et al. 1993) leads to the expectation that these mutations also negatively impact its growth.

Taken together, the observed pleiotropic growth patterns suggest that the signatures of architectural trade-offs can be detected in other resources, warranting further investigation. This would allow us to answer how such constraints manifest themselves dependent on the preadapted initial state of the population and how predictably we may observe evolutionary diversification in populations subject to resource-utilization trade-offs.

The metabolic adaptations observed in our evolution experiment manifested themselves at multiple levels of organization: as molecular changes in the organization and regulation of metabolic pathways (figs. 5 and 6), as differences in resource-utilization profile at the organismal level (fig. 4), and in the form of variation in ecological function (fig. 3). Linking these patterns enabled us to unravel how constraints interact with selection over the course of adaptive evolution and to develop an integrated understanding of the evolution of diversity in resource specialization. From our experiment, we see that historical contingency (e.g., preadaptation to glucose) was a crucial determinant to interpret the evolved adaptive mutations. It seems that within experimental evolution in general, there is bias toward focusing more on how selective pressure direct adaption rather than on how constraints obstruct it. Our study emphasizes that in order to understand evolutionary dynamics it is just as important to understand where you evolved from as where you evolve toward.

Interesting future work may include reevolving our galactose and fructose optimized specialists on glucose, mannose, and trehalose, and/or evolving the strains on different pairs of sugar combinations. Such experiments will provide additional insight into the generality and nature of the metabolic architectural constraints identified by our study. This would allow us to answer how such constraints manifest themselves dependent on the preadapted initial state of the population and how predictably we may observe evolutionary diversification in populations subject to resource-utilization trade-offs.

## Materials and Methods

### Experimental Procedures

Biofilm formation undermines the effectiveness of selection for increased growth rate in prolonged continuous cultures. Therefore, we used *L. cremoris* with its low biofilm forming properties instead of more commonly used model organisms. *Lactococcus cremoris* MG1363 (formerly called *Lactococcus lactis* subsp. *cremoris* MG1663) was grown in 60 ml of chemically defined medium for prolonged cultivation (CDMPC) (Price et al. 2019). Cultures were grown anaerobically (25 ml/min N_2_ headspace flow) under continuous dilution and stirring (330 rpm) at 30 °C and maintained at constant pH 6.5. Three treatments were run in parallel, using media supplemented with either 1% wt/v galactose, 0.5% wt/v fructose, or a mix of 0.5% wt/v galactose + 0.25% wt/v fructose as sole carbon source(s). For each of the three treatments, we ran four replicates in parallel (supplementary fig. S2, Supplementary Material online). The unequal amounts of fructose and galactose (1:2 wt/v ratio) that were provided in the culture media, compensated partially for the observed high asymmetry in growth rate of the ancestral *L. cremoris* on fructose versus galactose. In order to further balance the population sizes across treatments at the start of the evolution experiment, the initial dilution rate was set at 0.2 for the galactose control treatment, and at 0.3 for the fructose and mix treatments. Throughout the experiment, samples of 1.5 ml were drawn daily from each bioreactor to measure OD_600_ and preserve glycerol stocks. Whenever population densities increased beyond OD_600_ = 1.0 or decreased below OD_600_ = 0.5, the dilution rate of the bioreactors was adjusted daily to restore 0.5 < OD_600_ < 1.0. Given that the dilution rate corresponds to the growth rate of the culture (population) in steady state, we monitored its change throughout the experiment for signs of evolutionary adaptation, and selected three timepoints for subsequent phenotypic analysis accordingly (supplementary fig. S6, Supplementary Material online). Weekly samples were taken to check for infections on glucose-supplemented M17 agar plates. The experiment was run for 38–58 days depending on the treatment, corresponding to 549–1111 generations of evolution.

### Mutant Library Construction and Phenotypic Analysis Through Agar-Plate Analysis

After the evolution experiment was completed, a library of evolved strains was created to phenotypically characterize the evolved mutants in each treatment (supplementary fig. S2, Supplementary Material online). We focused on three timepoints strategically positioned along the evolutionary trajectory, which were chosen based on the observed growth-rate increase of each culture (see above). To analyze the phenotypes of the evolved strains, –80 °C glycerol population samples from the bioreactors were plated both on F-CDMPC (fructose) and G-CDMPC (galactose) agar plates. A sample from the glycerol stock was taken with a sterile toothpick and put into 1.5 ml PBS and subsequently diluted 500 times; 50 µl of the diluted cell suspension was then plated. Population samples from the fructose treatment were plated on F-CDMPC plates, those from the galactose treatment on G-CMDPC plates, and those from the mix treatment on both types of plate. After 48 h of incubation at 30 °C, the plates were photographed for further analysis. We picked six colonies per plate to construct a library of single genotypes from the population samples (i.e., 12 genotypes were sampled from each replicate population from the mix treatment, 6 genotypes from the fructose treatment, and 6 from the galactose treatment per timepoint). To sample the population-sample plates as broadly as possible for all occurring phenotypes, we chose to pick colonies of contrasting sizes from each plate: two large, two average-sized, and two small colonies (six in total). Each colony was then grown separately overnight at 30 °C without shaking in 2 ml F-CDMPS or G-CDMPC (depending on the treatment it derived from), and glycerol stocks were prepared from these cultures, yielding a library of 288 single genotype strains. These single-genotype glycerol stocks were then used to inoculate G-CDMPC and F-CDMPC plates, following the same plating procedure as for the population samples and also photographed. This replating step was important to characterize the performance of each evolved single-genotype in each sugar. The photographs from both the population-level plates (bioreactor samples) and single genotype plates (the picked colonies) were analyzed with the OpenCFU software to measure total colony count and area (pixel count) for each individual colony. In order to quantify the relative performance of the evolved single-genotypes on fructose versus galactose, we calculated the ratio between the total colony count on the F-CDMPC plate and on both plates combined (F-CDMPC + G-CDMPC); this (*F*/[*F* + *G*]) ratio was also computed for the median colony size.

### Growth Curves for Single Genotypes and Phenotypic Clustering Analysis

Growth rates on fructose and galactose were measured for all genotypes from the library (see above) after preculturing them overnight in F-CDMPC and G-CDMPC. The –80 °C stocks were diluted 100 times in PBS, 1 µl of this cell suspension was used to inoculate 100 µl of fresh fructose, galactose, and mixed sugar CDMCP (pH = 6.5). Each strain was grown in triplo in 384-well plates (*Greiner Bio-one 781906*) under semianaerobic conditions (*VIEWseal Greiner Bio-one*) at 30 °C, whereas keeping track of the OD_600_ in a plate reader (*Tecan F200*). After background correction, estimates of the instantaneous growth rate were obtained by performing local linear regression analyses on the ln-transformed growth curve data, using a sliding window of five data points (measurements were taken every 10 min) and OD_600_ > 0.16. We then determined the maximum growth rate from these regression curves and averaged the three-replicate maximum growth-rate values for each genotype on each sugar (see supplementary fig. S11, Supplementary Material online for example growth curves).

We used a model-based clustering method to identify and classify the different phenotypic groups that evolved during the evolution experiment (supplementary fig. S2, Supplementary Material online). The clustering was performed on the maximum growth rates of the single genotypes on fructose and galactose. The performance of each single genotype was plotted as a two-dimensional coordinate: *F*_max_ (maximum growth rate on fructose) on the *y*-axis and *G*_max_ (maximum growth rate on galactose) on the *x*-axis. The growth rate coordinates in the *F*_max_–*G*_max_ space were clustered per timepoint per treatment. To estimate the number of clusters (phenotypic groups), we applied normal (Gaussian) mixture models and a maximum likelihood approach using the R package *mclust* (version 5.4, Fraley et al. 2016). To consistently apply the same clustering model to all the treatments and timepoints we selected the EVV model, which allows clusters of ellipsoidal shape (i.e., bivariate Gaussian distributions) with different covariance structures (i.e., different orientations) (supplementary fig. S7, Supplementary Material online). This model was either the best model selected by the Bayesian Information Criterion (BIC, maximum likelihood corrected for model complexity) or yielded the same number of clusters as the best model selected by BIC independently for each timepoint and treatment. We limited the maximum number of clusters to three because we were only interested in the main phenotypic groups in the *F*_max_–*G*_max_ space (i.e., FS, GS, and generalist).

### DNA Sequencing and Analysis

We sequenced 48 strains by sampling three strains from two out of four replicates for each timepoint for each treatment. We set a threshold of only considering mutated loci that exclusively occurred in one phenotype (FS or GS) and which entailed five or more strains or variants. The isolation of chromosomal DNA of the population and single-genotype cultures was performed as described in Johansen and Kibenich (1992). Full-genome resequencing was performed using *IlluminaHiSeq* (*GATC*) with a mean coverage of 300, read length, and insert size of 150 bases. To characterize genetic variation across all strains, adaptors were removed with cutadapt 1.18 (Martin 2011), and pair reads were filtered and trimmed based on quality scores using trimmomatic 0.36 (Bolger et al. 2014) and FastQC 0.11.5. Filtered reads were then mapped to the reference genome (*Lactococcus lactis* subsp *cremoris* v. MG1363) using BWA 0.7.13 (Li and Durbin 2009). Duplicated reads were removed, and local realignment was performed using picard 2.18.5, increasing the maximum number of reads to 100,000 per base. Coverage values per base were calculated using SAMtools 1.9 (Li et al. 2009). Genotype calling was done with FreeBayes 0.9.10 (Garrison and Marth 2012), setting a minimum mapping quality of 20 and ploidy level of 1. This procedure results in a list of genetic variants that includes SNPs and indels. In order to consider only the genetic variants that resulted from the experiment, the genetic variants of each sample were contrasted with the variants observed in the ancestral strain (timepoint T0). Only the genetic variants that were observed in the evolved strains but not present in the ancestral strain were analyzed. In order to come up with a short list of loci that were specifically associated with either GS or FS adaptation, we identified a subselection of loci that most strongly correlated with fructose or galactose specialization. The criteria for identifying these loci were that they (1) occur exclusively in the fructose or galactose treatments, (2) have full annotation, and (3) feature SNP/indels in at least five strains or feature at least five variants (SNP/indel and CNV) (supplementary fig. S12, Supplementary Material online).

CNV along the genome was identified by measuring changes in local coverage relative to the flanking genomic regions and/or genome-wide coverage. CNV was inferred using CNVnator 0.3.3 (Abyzov et al. 2011), with a value of 100 for the parameter bin size and the option ‘unique’ in order to have the correct output of the quality field. With this method, no variants were detected in the ancestral strain. In general, for repetitive elements, the expectation is that a particular sample has the same number of copy fragments as in the reference genome. Thus, reads are expected to randomly map among reference copies in a uniform way, leading to a relative copy ratio between the sample and the reference of 1. CNV of a repeat will then lead to a variation in ratio around a value different from unity. As this variation will increase with the number of copies, the estimation of CNV between sample and reference becomes less accurate as the number of copies increases. However, since no CNVs were detected for the ancestral strain, CNVs found for repetitive sequences were considered to be biologically relevant (instead of a methodological artifact). With this method, we expect to accurately detect large changes in CNV, and likely miss events of small CNV change in particular for repetitive sequences.

To identify the potential phenotypic effect of the resulting list of genetic variants (SNPs, indels, and CNV) genetic variants were annotated relative to the reference genome using SnpEff 4.3 (Cingolani et al. 2012). Annotations for downstream, upstream, and interacting genes were not included. The 3D protein structure prediction from the *pfk* and *ldh* proteins was performed using *Phyre2* (Kelley et al. 2015). *EzMol* was used to visualize the proteins, color functional domains, and mutated regions (Reynolds et al. 2018). *Protter* was used to visualize the intermembranal mutations in the mannose PTS (Omasits et al. 2014).

### Expression Experiments

Strains were grown in 60 ml batch in the bioreactor system at pH 6.5, 30 °C in F-CDMPC or G-CDMPC. Culture samples were harvested midexponentially (OD_600_ ≈ 0.45) and the cell pellet was immediately frozen in liquid nitrogen. RNA extraction and cDNA preparation were performed in *duplo*. cDNA samples were used to run a qRT-pcr with custom primers for the selected genes (supplementary table S2, Supplementary Material online). The expression of housekeeping gene *glyA* was measured in parallel for all samples as a control to normalize background expression levels between samples.

### PFK Assays

Protein extracts were prepared from midexponentially harvested cells grown on F-CDMPC. Essays were performed with a *MyBioSource Phosphofructokinase microplate Assay kit (catalog#MBS8243182)* and executed as stated by the manufacturer’s protocol.

### Lactic Acid Assays

Cultures were grown on CDMPC supplemented by 0.5% sugar (glucose fructose, mannose, or galactose) and harvested midexponentially. Essays were performed on supernatant with a *Megazyme L-lactic acid assay kit* and performed as stated by the manufacturer’s protocol.

## Supplementary Material

Supplementary data are available at *Molecular Biology and Evolution* online.

## Supplementary Material

msac124_Supplementary_Data

## Data Availability

The data underlying this article that are not available in the article or in its online Supplementary Material will be shared on reasonable request to the corresponding author.

## Appendix: Constraints, trade-offs, and the eco-evolutionary theory of adaptive diversification

### Constraints Shape the Distribution of Phenotypes that can be Realized by Selection

Unconstrained adaptive evolution is expected to induce phenotypic change in the direction of the selection gradient, eventually leading to a maximization of fitness. This process can be visualized by a population evolving along a trajectory on a metaphorical phenotypic adaptive landscape that follows the direction of steepest ascent before converging eventually on a fitness peak (supplementary fig. S1*A*, Supplementary Material online). In reality, however, evolution is hardly ever unconstrained, preventing populations from following the steepest path to a fitness peak, limiting the rate of phenotypic evolution, or redirecting adaptive evolution toward suboptimal fitness peaks.

In the context of the fitness landscape metaphor, constraints manifest themselves as regions of phenotype space that are difficult to reach or altogether inaccessible. An important reason that such inaccessible regions exist is that organisms are subject to physical, biochemical, or thermodynamical laws and conservation principles that restrict the topology of the feasible phenotype space. The degree to which organisms are constrained by these fundamental principles is clearly illustrated by the success of flux-balance analysis and related constraint-based modeling approaches (Price et al. 2004), which have been shown capable of producing detailed and accurate predictions of cellular metabolic fluxes based on minimal information on input fluxes and the metabolic network in combination with a consistent treatment of basic conservation principles such as mass-, energy-, and redox-balance.

An equally important class of constraints is probabilistic ones, which exist when a particular adaptive phenotype is feasible but highly unlikely to evolve, given the current state of the population. One example of a probabilistic constraint occurs when a population is separated from a fitness peak by a valley. Since there is no accessible path of beneficial mutations with small effect sizes, evolution could only reach such a peak by a mutational leap to the other side of the valley. Because such a macro-mutation is statistically highly unlikely, access to the peak is constrained. A similar problem can occur on rugged fitness landscapes with a high degree of reciprocal sign epistasis. Here, certain adaptive phenotypes may rely on a beneficial combination of multiple mutations that are each deleterious in isolation, so that there is no gradual adaptive path toward the fitness peak (Dawid et al. 2010). Probabilistic constraints may also derive from the stochastic nature of mutation, demography, and gene fixation, which limit the rate of adaptive evolution in a finite population. Finally, it is common that certain (combinations) of phenotypes are more prone to mutate than others, or more variable due to a larger reservoir of standing genetic variation. These asymmetries are related to differences in the genetic or developmental architecture of traits, which may itself have been shaped by selection or reflect a long history of past adaptation. The short-term effect of architectural biases, however, is that evolution is accelerated in some directions in phenotype space, whereas it is inhibited in others, making it much harder for populations to reach certain adaptive peaks.

### Trade-offs and the Evolution of Specialist Strategies

By restricting access to a particular region of phenotype space, constraints can qualitatively alter the topology of the adaptive landscape. This effect is apparent in particular when organisms are prevented from optimizing multiple fitness components simultaneously (e.g., reproduction and survival), forcing them to find a balance between maximizing one fitness component at the cost of compromising the other. When such trade-offs between fitness components are strong (i.e., when fully maximizing one fitness component at the cost of the other yields a higher overall fitness than an intermediate compromise strategy), multiple alternative adaptive solutions tend to exist, reflected by the emergence of distinct peaks in the adaptive landscape (supplementary fig. S1*B*, Supplementary Material online).

Trade-offs readily emerge in two common situations. First, when a phenotypic character is important for two or more biological functions (i.e., when the trait is pleiotropic), it is unlikely that the optima for each individual function are located at the same position in the adaptive landscape. Optimizing the character for a given biological function then automatically leads to a suboptimal trait value for other functions. Second, trade-offs arise naturally in the context of allocation decisions. For example, the basic principle of energy conservation dictates that energy invested in a given biological function cannot be spent on another function, so that energetic investments necessarily trade-off against each other when the total budget is limited. Similarly, the allocation of metabolic fluxes to different metabolic pathways underlies a commonly observed trade-off between the rate and yield of microbial growth, given that alternative ATP producing pathways are all faced with a fundamental thermodynamic constraint, that is, a negative relationship between the yield and the rate of energy production.

### Adaptive Diversification and Frequency Dependance

Trade-offs figure prominently in the literature on adaptive diversification, because they are capable of generating multiple fitness peaks, corresponding to a disruptive selection regime. However, when acting on its own, disruptive selection tends to push populations toward one of the alternative fitness peaks, eventually depleting the available genetic variation (supplementary fig. S1*B*, Supplementary Material online). In order to prevent the rapid loss of polymorphism, adaptive diversification relies on one additional necessary condition (Metz et al. 1996; Geritz et al. 1998): in addition to being disruptive, it is critical that selection also be negatively frequency-dependent, that is, the success of each strategy must decline as it becomes more common.

When selection is frequency-dependent, thinking about the adaptive landscape as a static surface is misleading, because frequency dependance will cause the shape of the fitness landscape to change dynamically in response to any movement of the population in phenotype space (note that this is different from externally imposed fluctuating selection). Such dynamic feedback actually arises naturally in many ecological systems. Consider, for example, two genotypes that have evolved different resource-utilization strategies. When one of the two specialists becomes dominant in frequency, this will generally lead to a decrease in the concentration of the resources that it consumes, making its growth conditions less favorable. The other specialist might be affected by the decreased resource availability as well, but to a lesser extent, because it has at least partially specialized on a different set of resources, which are not consumed as much by the dominant type. As a result, the rare specialist enjoys a relative fitness advantage that will allow it to increase in frequency relative to the dominant type. If the resource-utilization profiles are sufficiently different, the frequency effect can be so strong that the two specialists are driven toward an ecological coexistence equilibrium, where their fitness values are dynamically maintained to be equal (supplementary fig. S1*C*, Supplementary Material online).
