# Supplementary material for: Trade-Offs Predicted by Metabolic Network Structure Give Rise to Evolutionary Specialization and Phenotypic Diversification
Source: Mol Biol Evol. 2022 Jun 9;39(6):msac124. doi: 10.1093/molbev/msac124 (PMC9206417; doi:10.1093/molbev/msac124)
Supplement: msac124_Supplementary_Data [file msac124_supplementary_data.zip › Ekkers_et-al_MBE_online supplementary material_final.pdf]

## SUPPLEMENTARY MATERIAL

## Ekkers *et al.* Trade-offs predicted by metabolic network structure give rise to evolutionary specialization and phenotypic diversification

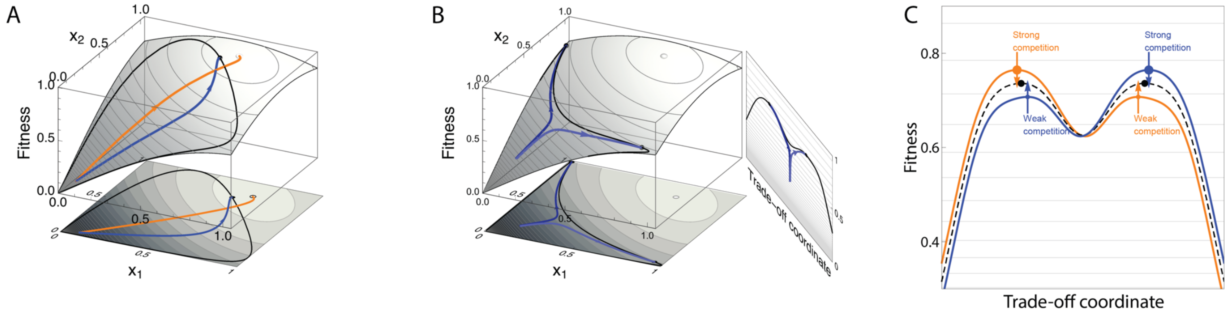


**Supplementary Figure S1 - Key concepts in the eco-evolutionary theory of adaptive diversification. (A)** The 3D-surface plot depicts fitness as a function of two phenotypic traits, $x_1$ and $x_2$, but is meant as a simplified metaphor for the dependence of fitness on a large number of phenotypic characters. Slight phenotypic variations, which are continually introduced into the population by mutation, tend to become established in the population if they lead to an increase in fitness. In the absence of any constraints, the mean phenotype in the population therefore evolves in the direction of the steepest gradient on the fitness surface (orange trajectory), to end up eventually at a fitness maximum (light-grey circle). Constraints can interfere with the action of selection by restricting access to certain combinations of phenotypes or by biasing the variation that is introduced by mutation. As illustrated by the blue trajectory, constrained evolution no longer necessarily follows the direction of steepest ascent and may converge on a suboptimal final phenotype; here, the final outcome is constrained by a phenotypic trade-off (indicated by the black line). The projection of the 3D-plot on the phenotype space shows that the trade-off in (A) is weak, i.e., the trade-off curve is concave relative to the fitness contours. In **(B)**, by contrast, the trade-off is strong, creating two alternative (constrained) optimal phenotype combinations (black dots). Evolutionary trajectories (light and dark blue) now converge to either one of the fitness maxima (depending on initial conditions and chance effects), and the fitness landscape along the trade-off curve features two peaks (projection on the right). **(C)** Strong trade-offs do not necessarily support adaptive diversification, but rather tend to favor the evolution of a single specialist. Diversity can be maintained, however, when selection is frequency dependent. The three curves illustrate the dynamics of the fitness landscape due to negative frequency dependence in a polymorphic population consisting of two ecological specialists located at either end of the trade-off curve (dots). When one ecological specialist has a higher fitness than the other (blue or orange curve), its relative frequency will increase, inducing more severe competition, which subsequently reduces its fitness value. As a result of this negative feedback, the two specialists are dynamically maintained in an ecological equilibrium at which their fitness values are equal (black dotted line). See Appendix for further details.

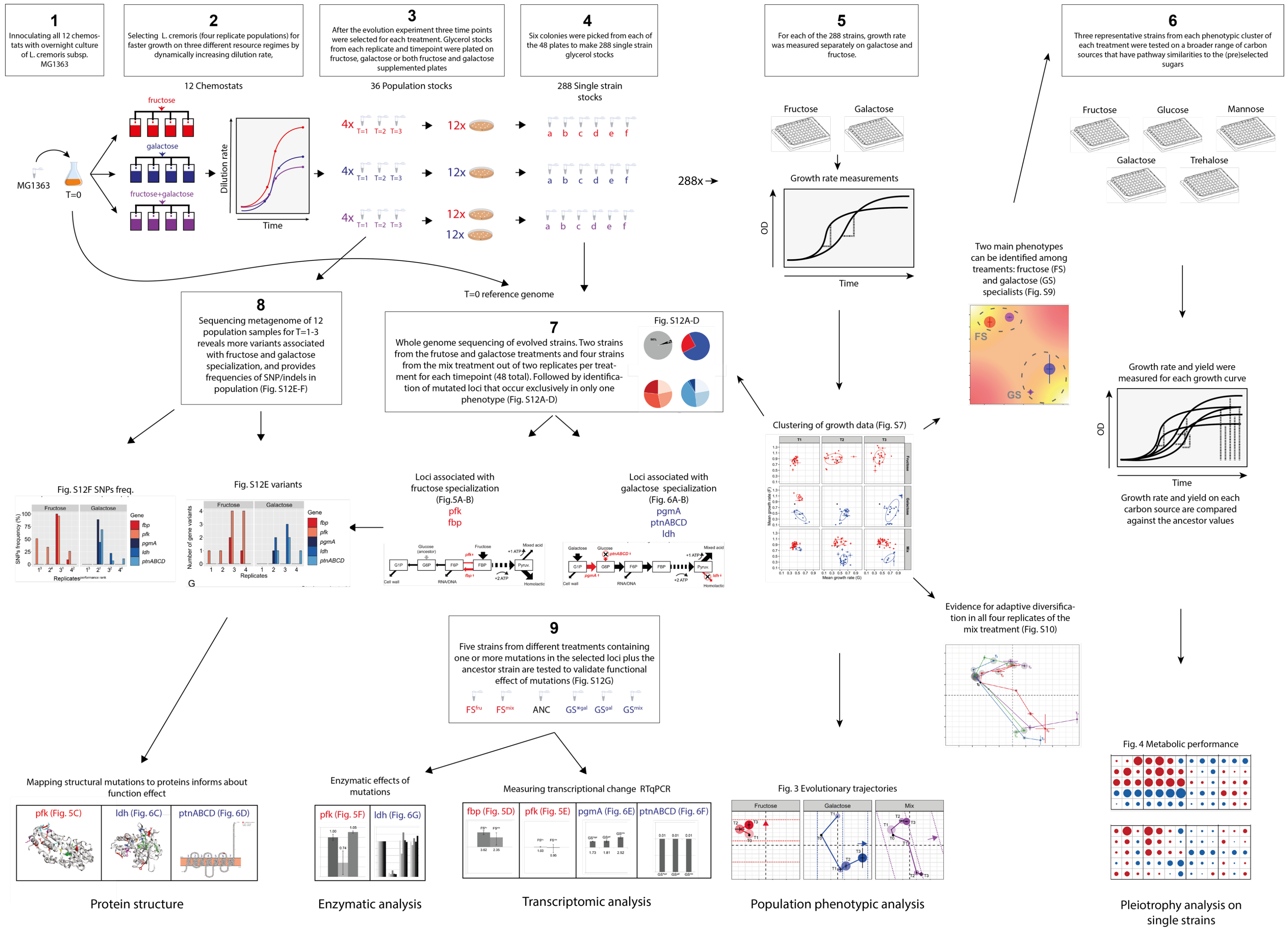


**Supplementary figure S2** - Flowchart of the methods and results. The chronology of the analyses are indicated by numbers 1-9. The interconnectivity and dependency of analyses in terms of information and workflow is further indicated with arrows. Results of analyses are referenced by figure names.

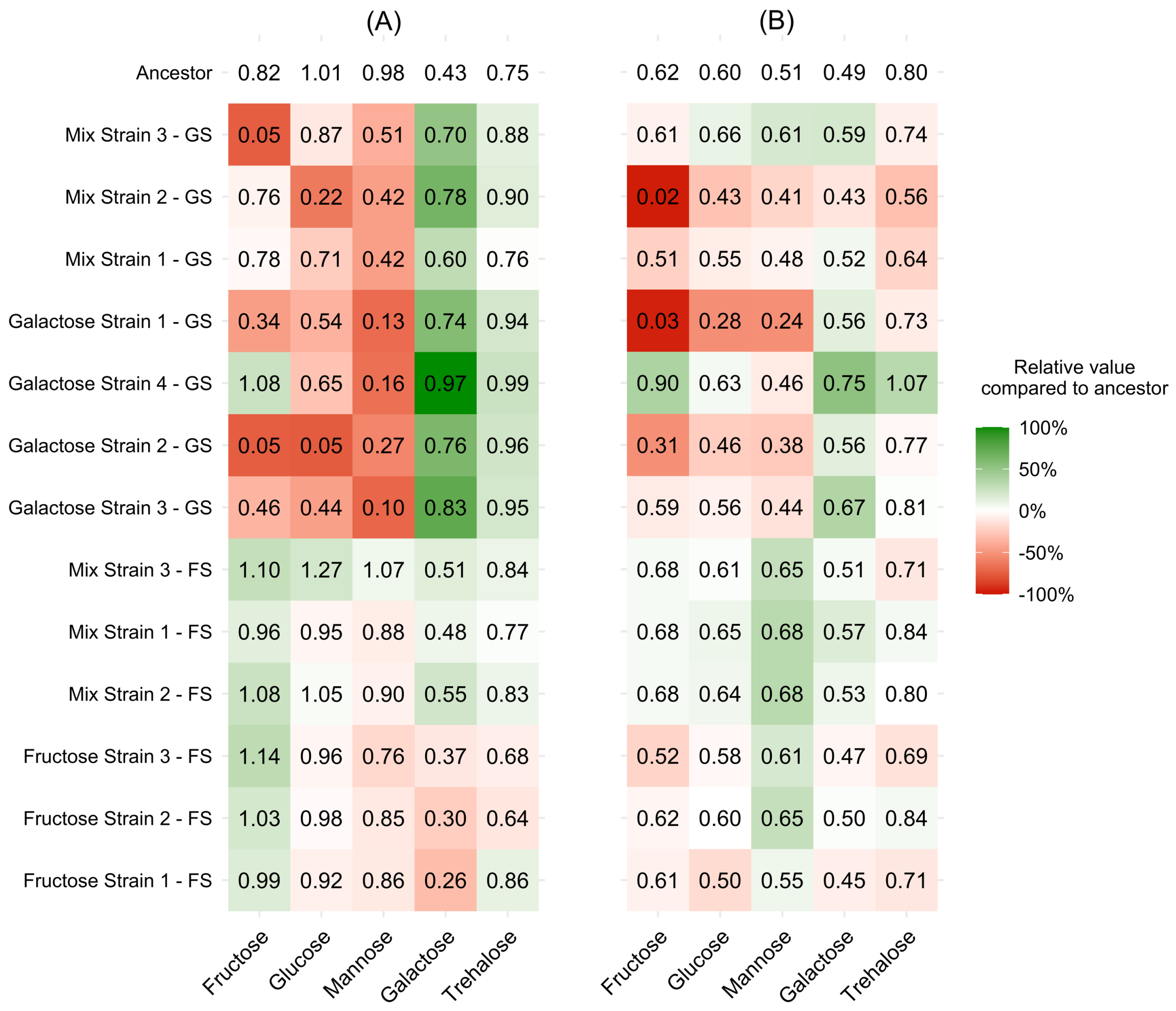


| (A) | Fructose | Glucose | Mannose | Galactose | Trehalose |
|---|---|---|---|---|---|
| Ancestor | 0.82 | 1.01 | 0.98 | 0.43 | 0.75 |
| Mix Strain 3 - GS | 0.05 | 0.87 | 0.51 | 0.70 | 0.88 |
| Mix Strain 2 - GS | 0.76 | 0.22 | 0.42 | 0.78 | 0.90 |
| Mix Strain 1 - GS | 0.78 | 0.71 | 0.42 | 0.60 | 0.76 |
| Galactose Strain 1 - GS | 0.34 | 0.54 | 0.13 | 0.74 | 0.94 |
| Galactose Strain 4 - GS | 1.08 | 0.65 | 0.16 | 0.97 | 0.99 |
| Galactose Strain 2 - GS | 0.05 | 0.05 | 0.27 | 0.76 | 0.96 |
| Galactose Strain 3 - GS | 0.46 | 0.44 | 0.10 | 0.83 | 0.95 |
| Mix Strain 3 - FS | 1.10 | 1.27 | 1.07 | 0.51 | 0.84 |
| Mix Strain 1 - FS | 0.96 | 0.95 | 0.88 | 0.48 | 0.77 |
| Mix Strain 2 - FS | 1.08 | 1.05 | 0.90 | 0.55 | 0.83 |
| Fructose Strain 3 - FS | 1.14 | 0.96 | 0.76 | 0.37 | 0.68 |
| Fructose Strain 2 - FS | 1.03 | 0.98 | 0.85 | 0.30 | 0.64 |
| Fructose Strain 1 - FS | 0.99 | 0.92 | 0.86 | 0.26 | 0.86 |

| (B) | Fructose | Glucose | Mannose | Galactose | Trehalose |
|---|---|---|---|---|---|
| Ancestor | 0.62 | 0.60 | 0.51 | 0.49 | 0.80 |
| Mix Strain 3 - GS | 0.61 | 0.66 | 0.61 | 0.59 | 0.74 |
| Mix Strain 2 - GS | 0.02 | 0.43 | 0.41 | 0.43 | 0.56 |
| Mix Strain 1 - GS | 0.51 | 0.55 | 0.48 | 0.52 | 0.64 |
| Galactose Strain 1 - GS | 0.03 | 0.28 | 0.24 | 0.56 | 0.73 |
| Galactose Strain 4 - GS | 0.90 | 0.63 | 0.46 | 0.75 | 1.07 |
| Galactose Strain 2 - GS | 0.31 | 0.46 | 0.38 | 0.56 | 0.77 |
| Galactose Strain 3 - GS | 0.59 | 0.56 | 0.44 | 0.67 | 0.81 |
| Mix Strain 3 - FS | 0.68 | 0.61 | 0.65 | 0.51 | 0.71 |
| Mix Strain 1 - FS | 0.68 | 0.65 | 0.68 | 0.57 | 0.84 |
| Mix Strain 2 - FS | 0.68 | 0.64 | 0.68 | 0.53 | 0.80 |
| Fructose Strain 3 - FS | 0.52 | 0.58 | 0.61 | 0.47 | 0.69 |
| Fructose Strain 2 - FS | 0.62 | 0.60 | 0.65 | 0.50 | 0.84 |
| Fructose Strain 1 - FS | 0.61 | 0.50 | 0.55 | 0.45 | 0.71 |

**Supplementary Figure S3 – Metabolic performance profile of selected fructose and galactose specialists (FS and GS, respectively).** Evolved strains were grown in triplicates in batch on fructose, glucose, mannose, galactose or trehalose (1% wt/v) supplemented CDMPC. Values indicate absolute maximum growth rate (A) or biomass yield (B), and colors indicate of the percent increase (green) or decrease (red) compared to the ancestral strain (first row). Evolved strains were grown in triplicates in batch on fructose, glucose, mannose, galactose or trehalose (1% wt/v) supplemented CDMPC.

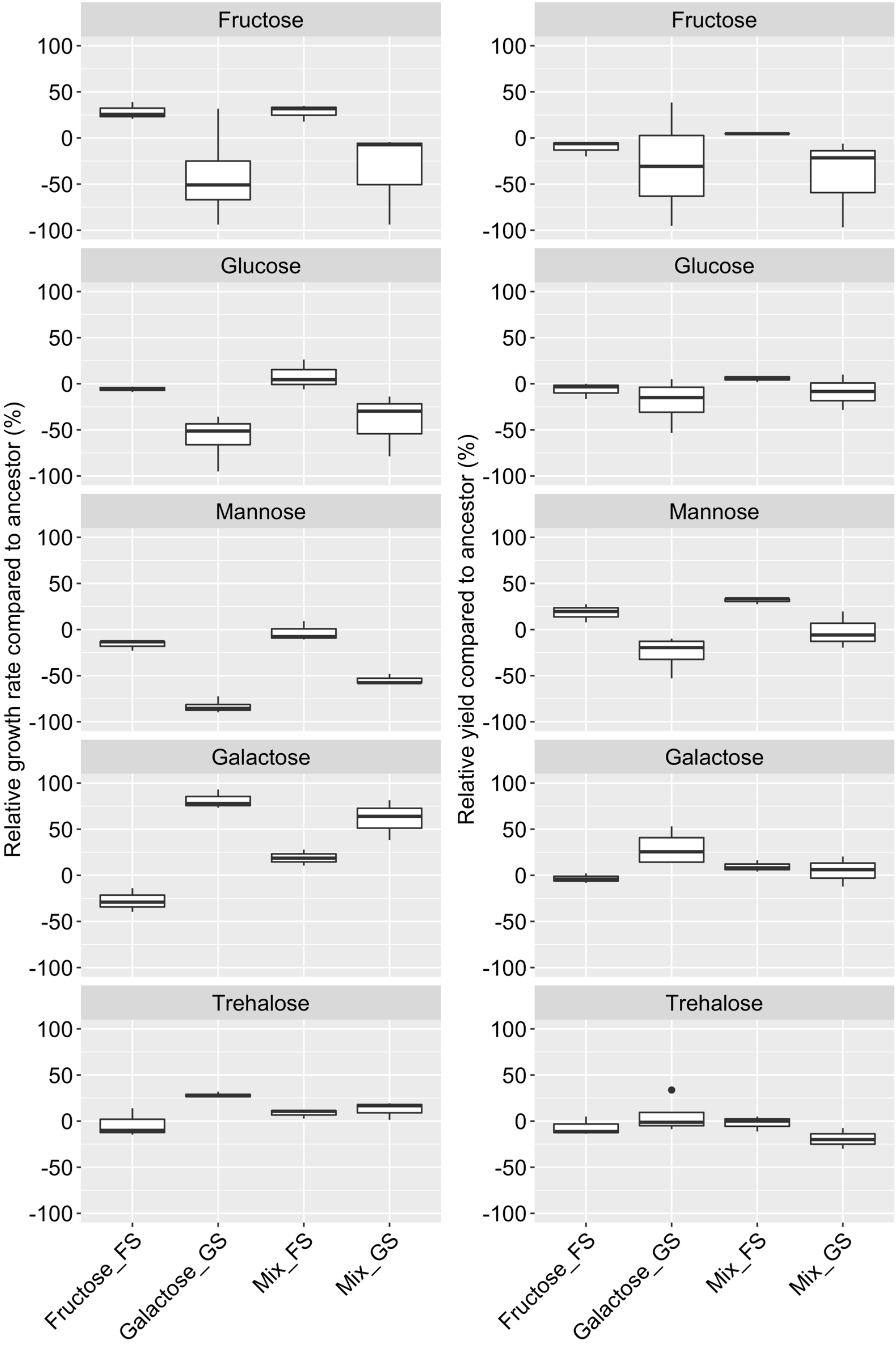


**Supplementary Figure S4 -** Distribution of the relative maximum growth rate (left) and yield (right) of the evolved strains compared to the ancestor (see also Supp. Fig. S3). The evolved strains are classified as fructose specialist (FS) or galactose specialist (GS) in the three treatments (fructose only, galactose only and mixed). Boxplots that cross the zero line indicate that the change in growth and/or yield of the evolved strain was not significantly different than the ancestor.

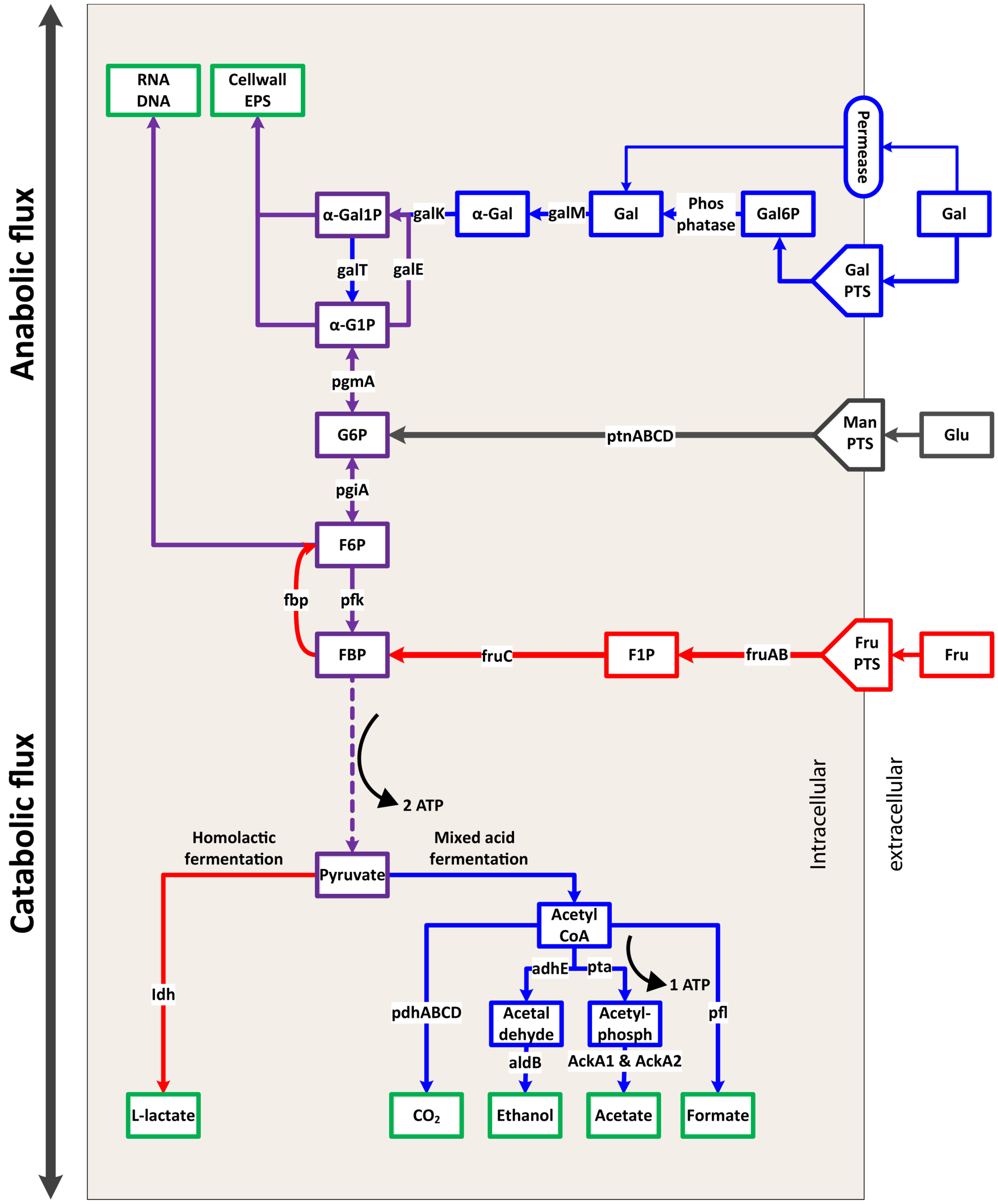


**Supplementary Figure S5 – Schematic representation of the metabolic architecture of the central carbon metabolism of *L. lactis subsp. cremoris* MG1363.** The metabolic fluxes are visualized by color coded arrows indicating the net direction of metabolic flux of galactose (blue) and fructose (red) specific pathways and shared pathways (purple), text on the arrows indicate gene names of the enzymes related to each metabolic step. The entry point of the ancestrally preadapted sugar (glucose) is indicated in grey. Metabolites are shown as squares.

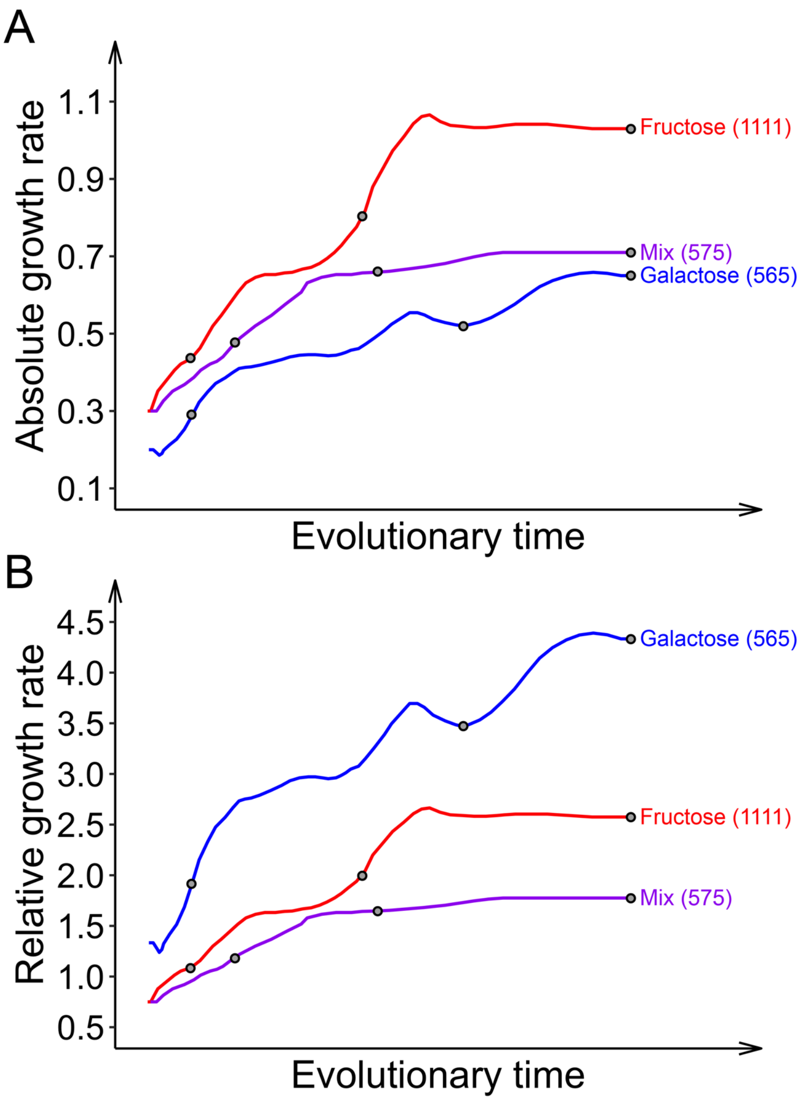


**Supplementary Figure S6 – Adaptive trajectories for the three experimental groups.** (A) Population growth rate increases during the evolution experiment for the galactose (blue), fructose (red) and mix (purple) treatment along with adjustments of the bioreactor dilution rate over the course of prolonged cultivation in turbidostat (manually-controlled density) mode. The x-axis in both figures reflects the number of generations for each treatment from the start to the end of the experiment (due to growth rate differences, the scaling is different between treatments; total number of generations is indicated at the end of each line). The dots on each line in both graphs indicate the sample timepoints T1, T2 and T3. The upper and lower panel show, respectively, the absolute growth rate and the growth rate relative to the performance of the ancestral strain under each of the treatment conditions.

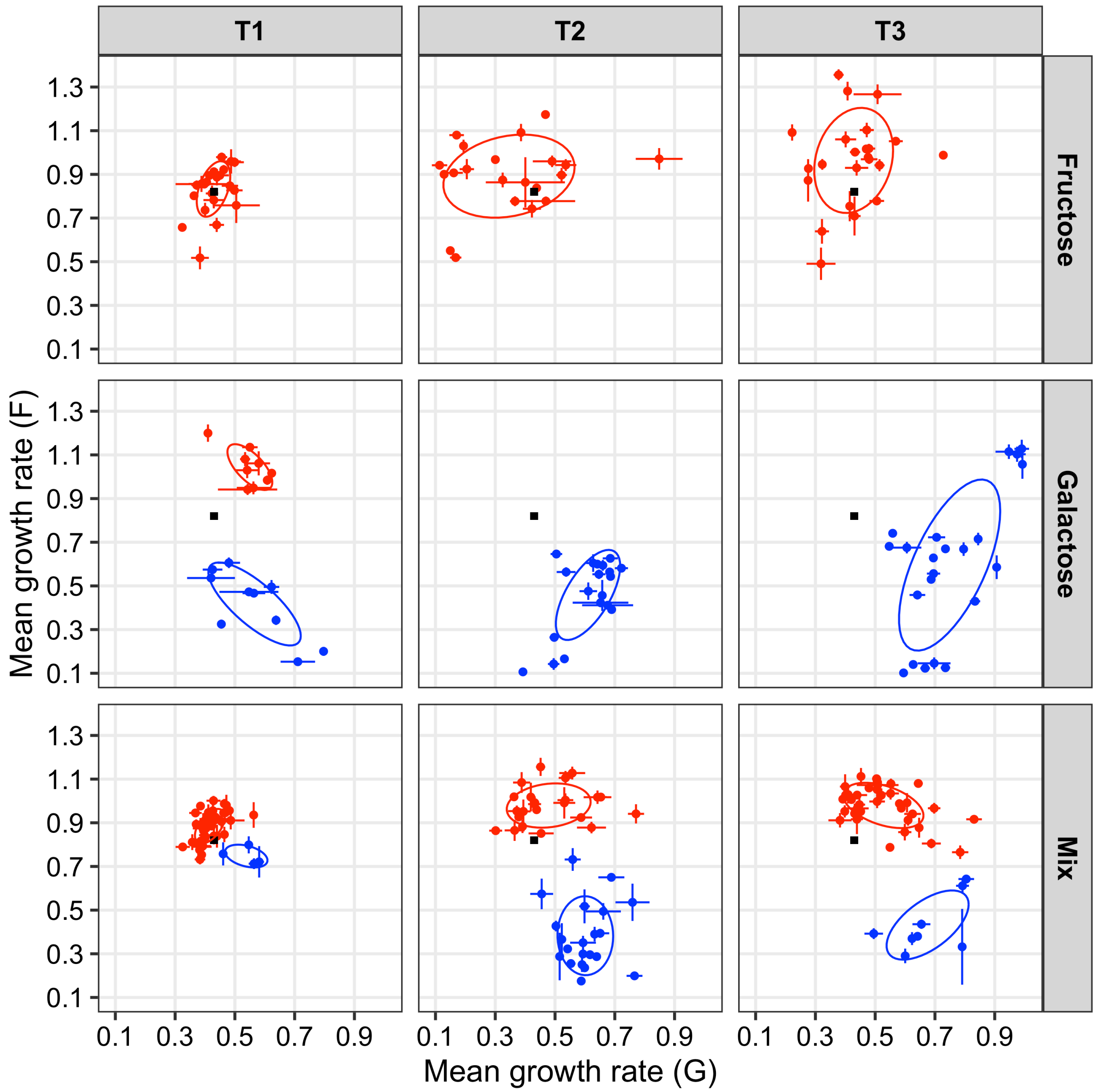


**Supplementary Figure S7 – Phenotypic clustering analysis.** Clustering was performed on the maximum growth rate coordinates on fructose (F) and galactose (G) for each of the genotypes sampled from the evolving populations across the three treatments (fructose only, galactose only and mix treatments; rows) at timepoint T1, T2 and T3 (columns). The two colors indicate the different phenotypic clusters. Each strain (dot) was measured three times for growth (see Supp. Fig. S11); error bars indicate these multiple measurements. Note that in the Galactose treatment, at T3, there was the evolution in one replicate of strains with high mean growth rate on both sugars (i.e., dots on the top-left corner of the plot).

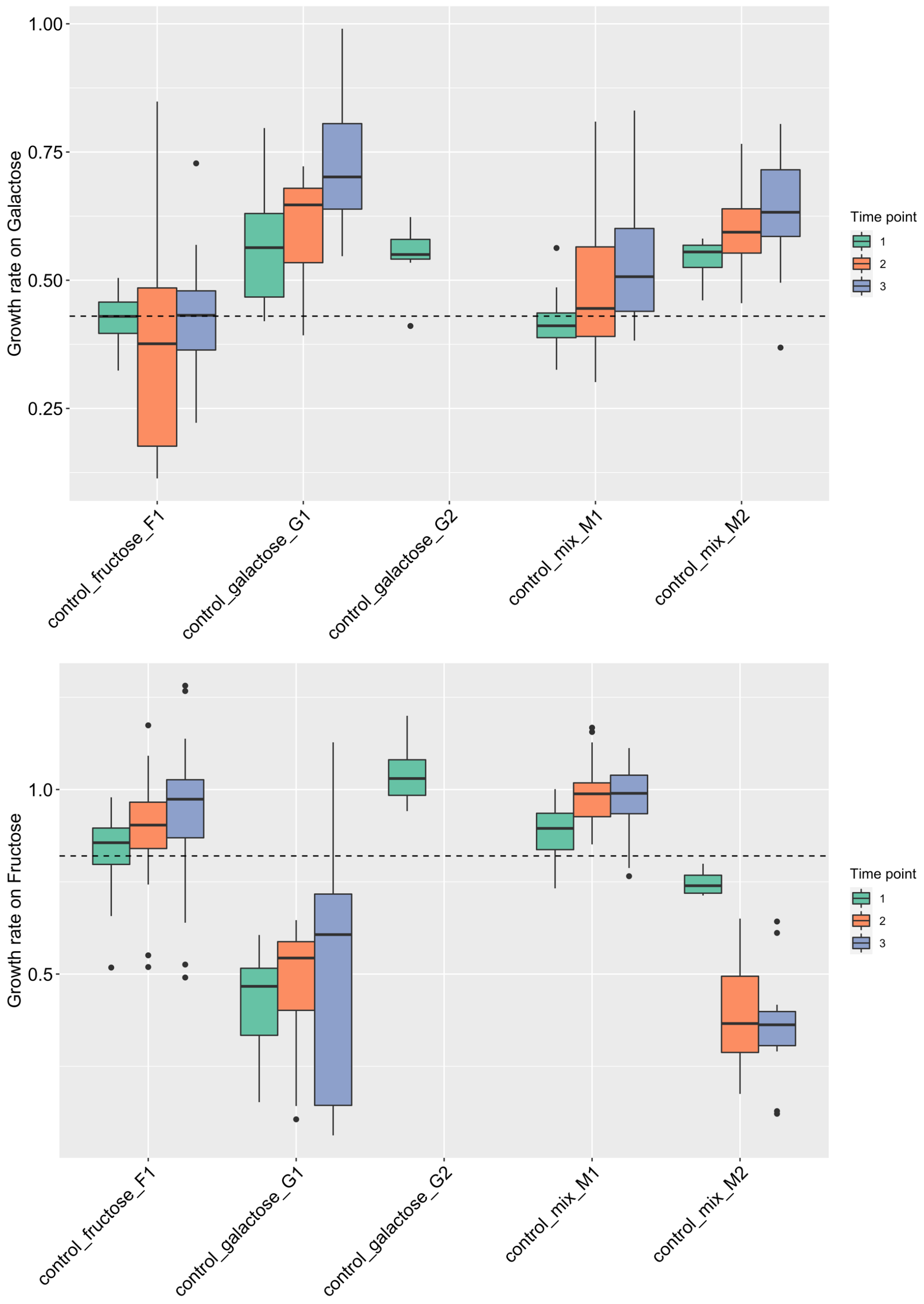


**Supplementary Figure S8 –** Boxplots of the maximum growth rate on galactose (top graph) and on fructose (bottom graph) for each of the genotypes sampled from the evolving populations across the clusters of the three treatments (fructose, galactose, mixed) at timepoint 1, 2 and 3 (colors of boxplots). For clusters of each treatment at each timepoint see Supp. Fig. S7. Note that the galactose treatment evolved two phenotypic clusters only at timepoint 1 (G1 and G2) whereas the mixed treatment evolved two clusters at the three timepoints (M1 and M2).

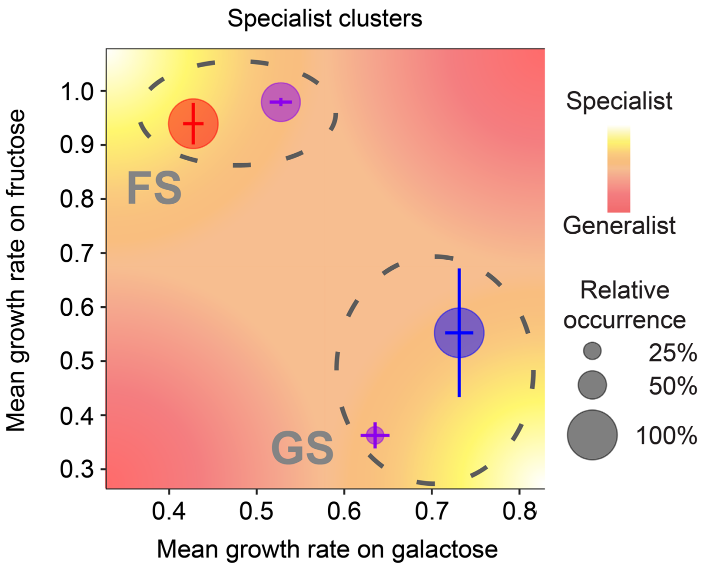


**Supplementary Figure S9 – Phenotypic clusters detected at timepoint T3 for fructose (red), galactose (blue) and mix (purple) treatments.** Dotted circles indicate phenotypic clusters that are either considered to be FS or GS. Error bars indicate SD.

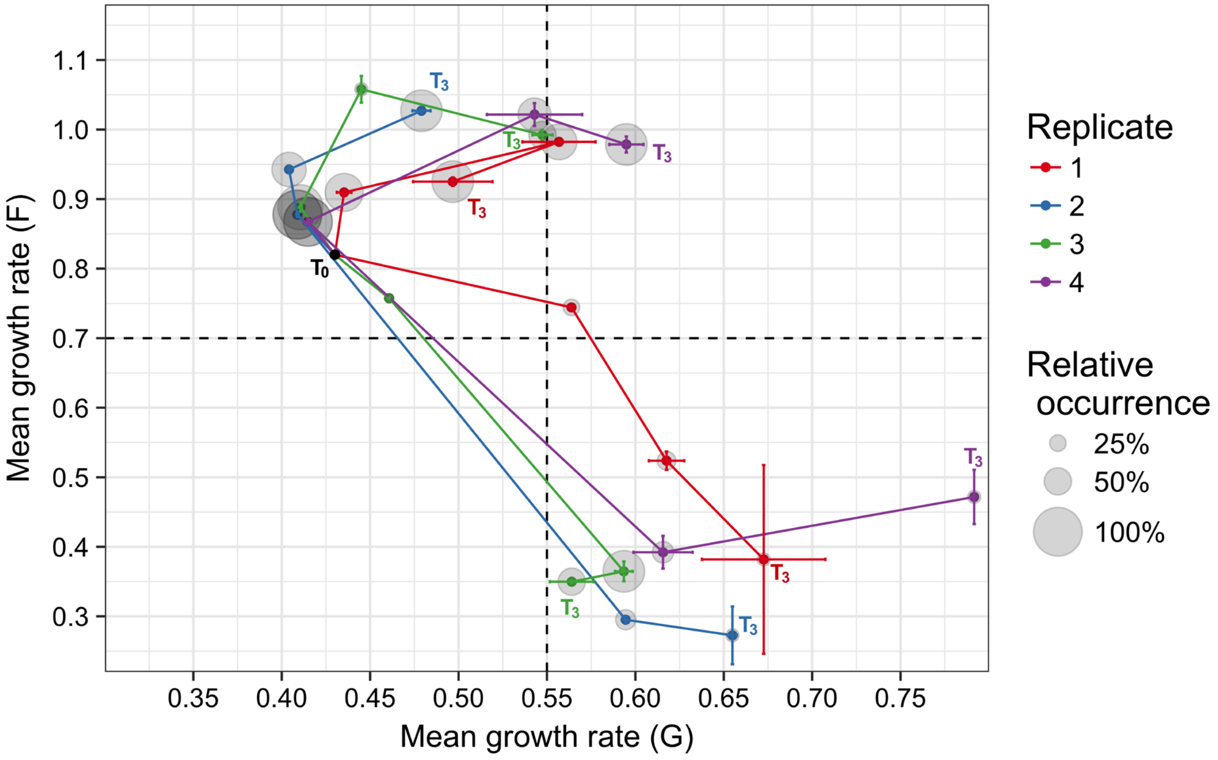


**Supplementary Figure S10 – Evolutionary trajectories of the separate replicate populations from the mix treatment.** The four replicate populations from the mix treatment (shown in different colors) show parallel phenotypic diversification.

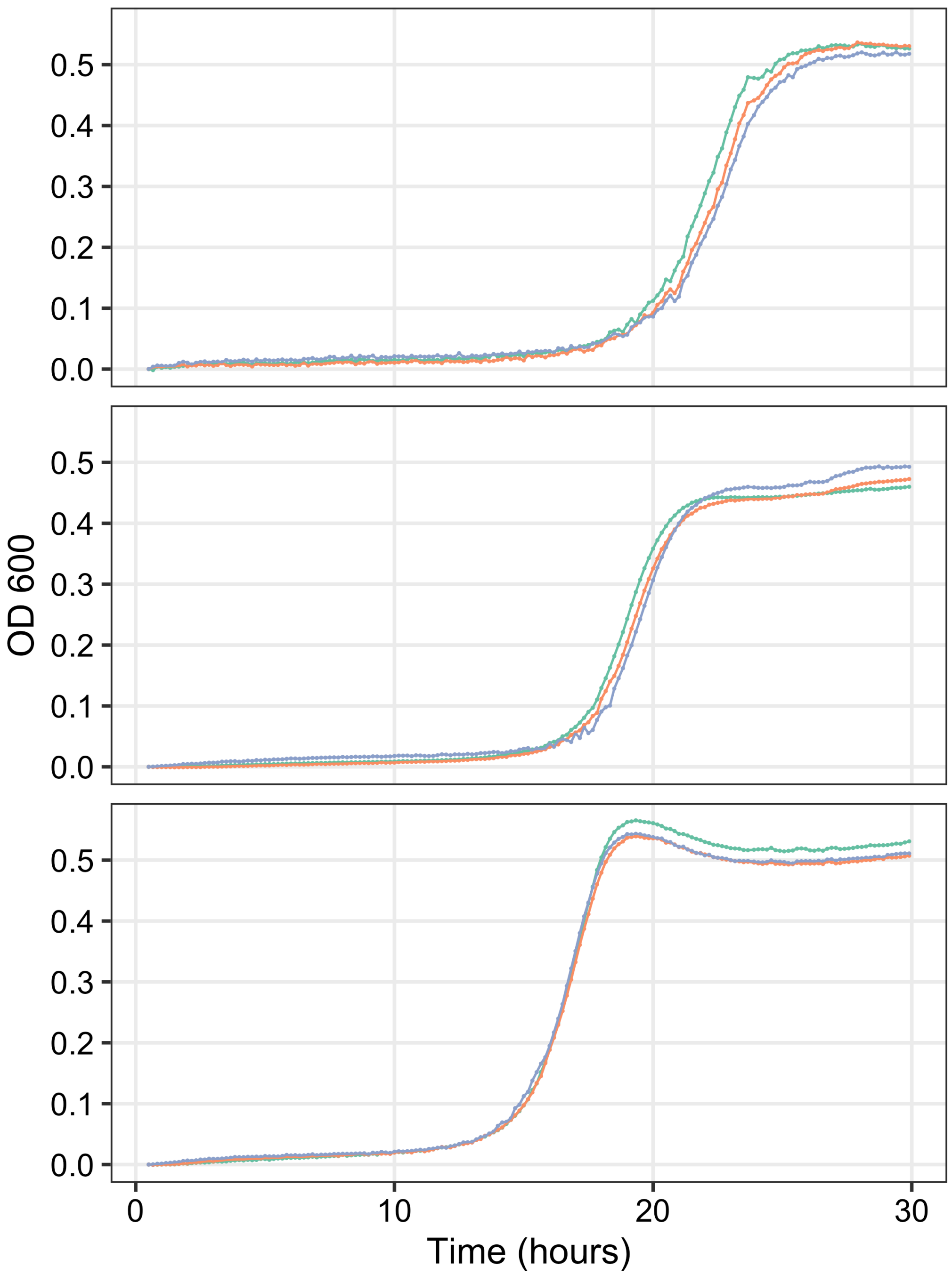


**Supplementary Figure S11** – Example ODs. Each graph shows a strain with three (triplicates) growth curves.

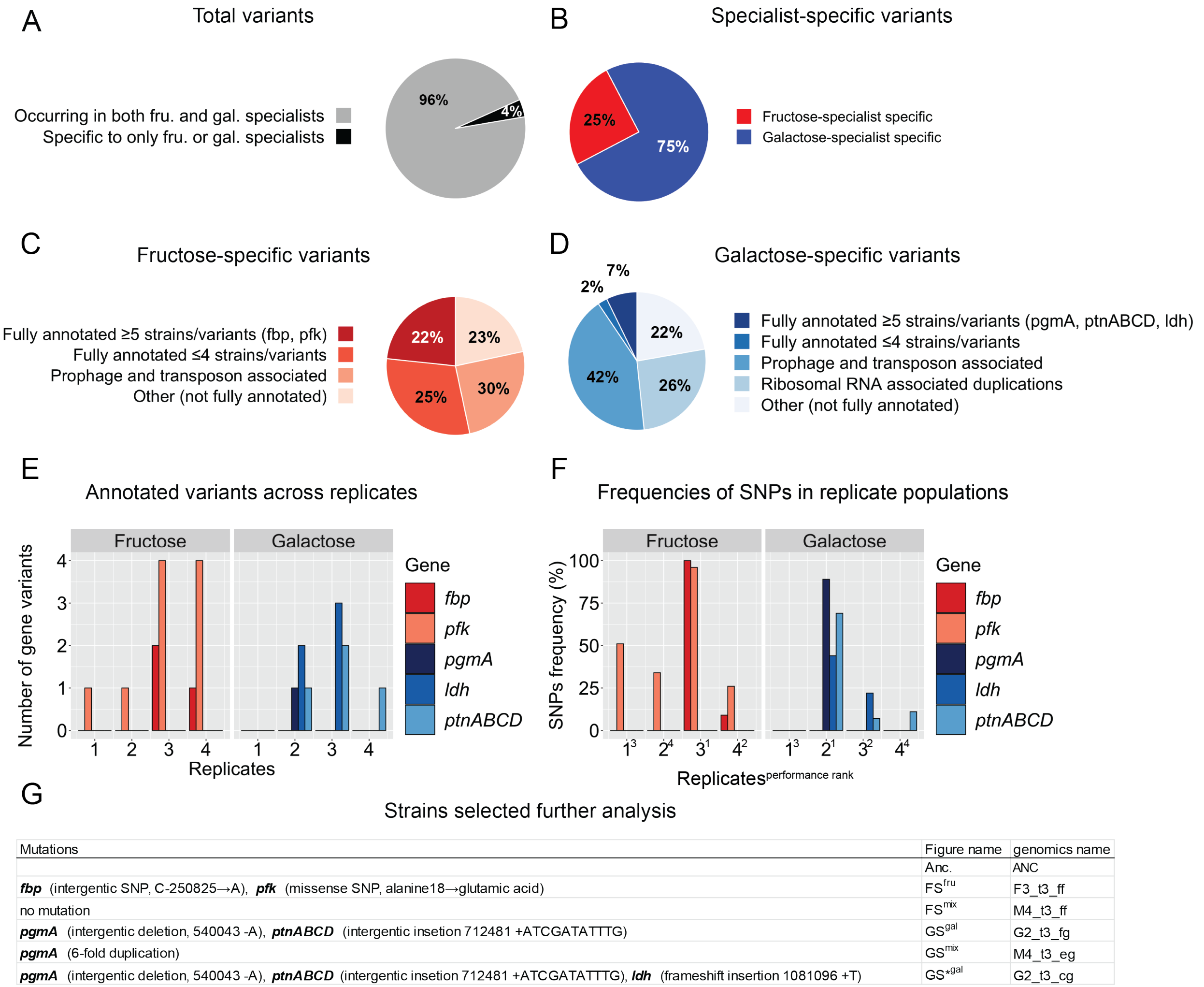


G

Strains selected further analysis

| Mutations | Figure name | genomics name |
|---|---|---|
| | Anc. | ANC |
| ***fbp*** (intergentic SNP, C-250825→A), ***pfk*** (missense SNP, alanine18→glutamic acid) | $FS^{fru}$ | F3_t3_ff |
| no mutation | $FS^{mix}$ | M4_t3_ff |
| ***pgmA*** (intergentic deletion, 540043 -A), ***ptnABCD*** (intergentic insetion 712481 +ATCGATATTTG) | $GS^{gal}$ | G2_t3_fg |
| ***pgmA*** (6-fold duplication) | $GS^{mix}$ | M4_t3_eg |
| ***pgmA*** (intergentic deletion, 540043 -A), ***ptnABCD*** (intergentic insetion 712481 +ATCGATATTTG), ***ldh*** (frameshift insertion 1081096 +T) | $GS*^{gal}$ | G2_t3_cg |

**Supplementary Figure S12 – Categorization of mutations.** (A) Distribution of mutations that were found exclusively in FS or GS across treatments. (B) Distribution of FS and GS specific mutations. (C) FS mutated loci. (D) GS mutated loci. (E) Occurrence of variants of the selected genes across fructose and galactose treatments derived from single genotype and population sequences. (F) Frequencies of SNPs in selected genes from the population sequences per replicate in the fructose and galactose treatments. Replicates are ranked for each treatment (ranking is indicated in superscript). The ranking is based on the average maximum growth rate of each replicate population on the selected sugar. (G) Strains selected for subsequent expression and enzymatic analysis.

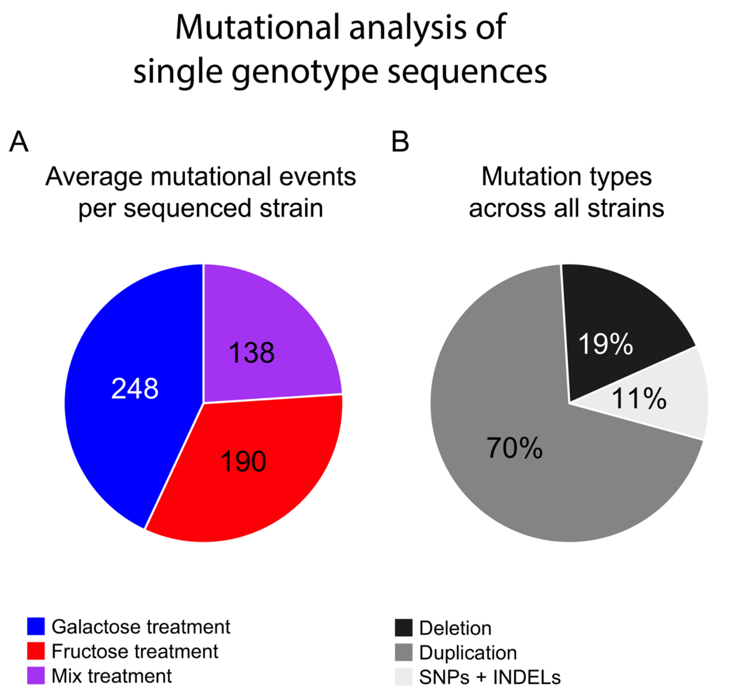


**Supplementary Figure S13 –** Mutational analysis of all single genotype sequences.

**Supplementary Table S2** – Primer sequences of qRT-PCR expression experiments

| Primer | Sequence |
|---|---|
| glyA_FW | ATGGGAAAGAAGCACAGGACTTATTAG |
| glyA_RV | TGCCGCACCAATTCGCAC |
| fbp_FW | TCGCAACTACAAAATCTGGGAGAAC |
| fbp_RV | CGCGGTCATAAATATCCCCAACAAC |
| pfk_FW | ACATTCTTGTACTCAGCACGTTACC |
| pfk_RV | CACCACCGATTACAACGACACC |
| pgmA_FW | TGTTGCTCAAGGAACTCAATATTATGCTC |
| pgmA_RV | AGCTGCTTTTTCAAGTGCTCCC |
| ptnAB_FW | AAATTGAAGCTGCCATCGCAAC |
| ptnAB_RV | TGGATTTTCACCCATCACTGCAC |

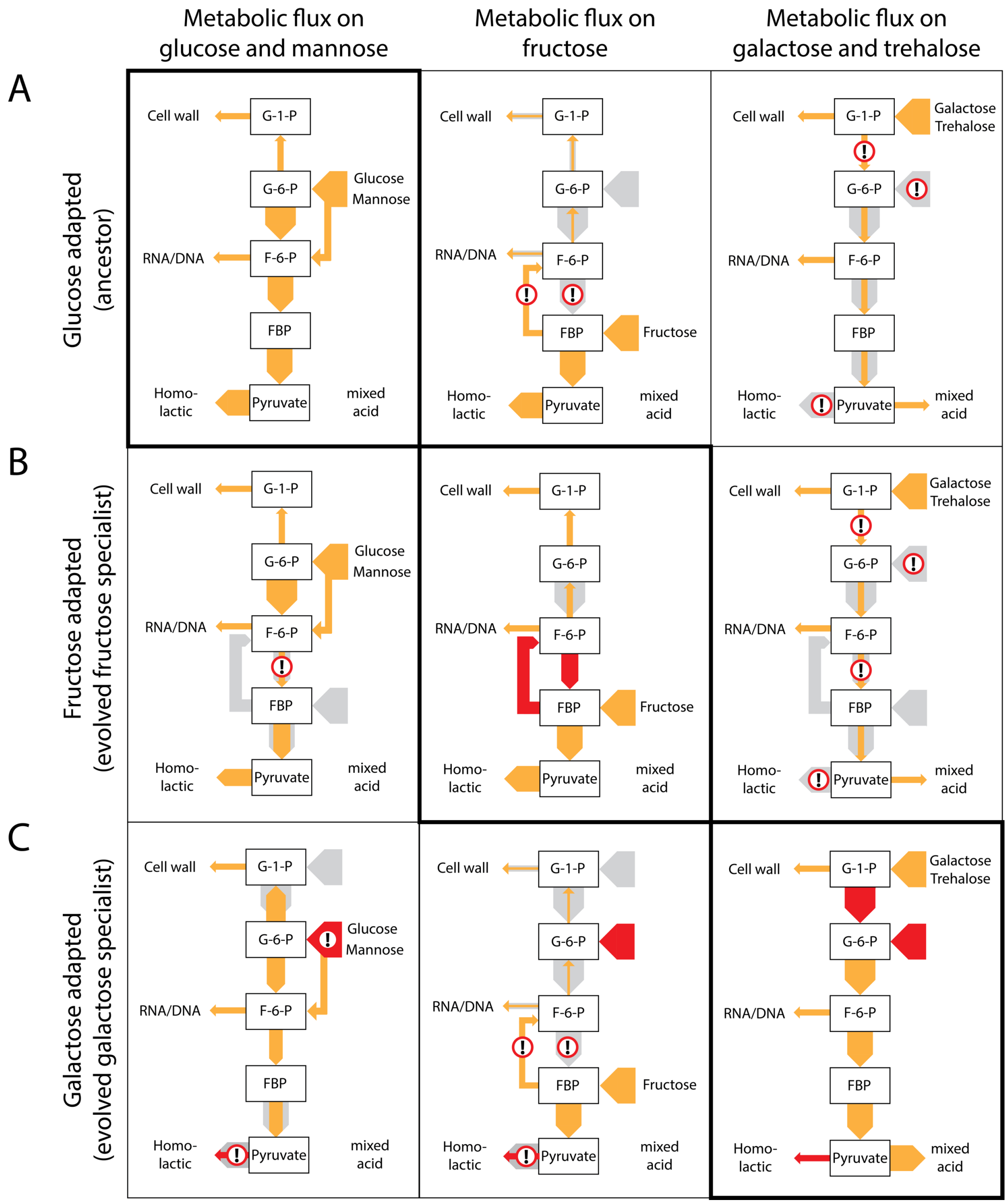


**Supplementary Figure S14 – Integrative model and functional predictions.** Diagrams show schematic representations of the architecture of glycolysis with sugar import points and catabolic (homolactic or mixed acid metabolism) and anabolic (cell wall, EPS, RNA and DNA synthesis) exit points. Arrows indicate the direction of metabolic fluxes, with relative widths suggests a flux rate. Fluxes that are active while metabolizing a specific sugar are shown in orange; grey arrows indicate the flux potential characteristic of adapted metabolic networks in glucose, fructose or galactose specialists; red arrows indicate enzymatic steps that have been mutated in the evolution experiment. Exclamation marks indicate predicted metabolic conflicts between adapted state and exposed sugar. The three columns show the predicted metabolic fluxes when exposed to glucose/mannose, fructose or galactose/trehalose (from left to right). The rows display glucose adapted ancestral state, fructose adapted state and galactose adapted state (top to bottom).
